# The Asymmetric Binding of PGC-1α to the ERRα and ERRγ Nuclear Receptor Homodimers Involves a Similar Recognition Mechanism

**DOI:** 10.1371/journal.pone.0067810

**Published:** 2013-07-09

**Authors:** Maria Takacs, Maxim V. Petoukhov, R. Andrew Atkinson, Pierre Roblin, François-Xavier Ogi, Borries Demeler, Noelle Potier, Yassmine Chebaro, Annick Dejaegere, Dmitri I. Svergun, Dino Moras, Isabelle M. L. Billas

**Affiliations:** 1 Department of Integrative Structural Biology, Institute of Genetics and Molecular and Cellular Biology (IGBMC), Centre National de la Recherche Scientifique (CNRS), UMR 7104, Institut National de la Santé et de la Recherche Médicale (INSERM) U964, Université de Strasbourg (UdS), Illkirch, France; 2 European Molecular Biology Laboratory, Hamburg Outstation, EMBL DESY, Hamburg, Germany; 3 Centre for Biomolecular Spectroscopy and Randall Division of Cell and Molecular Biophysics, King’s College London, London, United Kingdom; 4 SOLEIL Synchrotron, L'Orme des Merisiers Saint-Aubin, Gif-sur-Yvette, France; 5 NanoTemper Technologies GmbH, Munich, Germany; 6 Department of Biochemistry, University of Texas Health Science Center, San Antonio, Texas, United States of America; 7 Institut de Chimie LC3, Université de Strasbourg, Centre National de la Recherche Scientifique (CNRS), UMR 7177, Strasbourg, France; 8 INRA-URBIA, Nantes, France; Institut de Génomique Fonctionnelle de Lyon, France

## Abstract

**Background:**

PGC-1α is a crucial regulator of cellular metabolism and energy homeostasis that functionally acts together with the estrogen-related receptors (ERRα and ERRγ) in the regulation of mitochondrial and metabolic gene networks. Dimerization of the ERRs is a pre-requisite for interactions with PGC-1α and other coactivators, eventually leading to transactivation. It was suggested recently (Devarakonda *et al*) that PGC-1α binds in a strikingly different manner to ERRγ ligand-binding domains (LBDs) compared to its mode of binding to ERRα and other nuclear receptors (NRs), where it interacts directly with the two ERRγ homodimer subunits.

**Methods/Principal Findings:**

Here, we show that PGC-1α receptor interacting domain (RID) binds in an almost identical manner to ERRα and ERRγ homodimers. Microscale thermophoresis demonstrated that the interactions between PGC-1α RID and ERR LBDs involve a single receptor subunit through high-affinity, ERR-specific L3 and low-affinity L2 interactions. NMR studies further defined the limits of PGC-1α RID that interacts with ERRs. Consistent with these findings, the solution structures of PGC-1α/ERRα LBDs and PGC-1α/ERRγ LBDs complexes share an identical architecture with an asymmetric binding of PGC-1α to homodimeric ERR.

**Conclusions/Significance:**

These studies provide the molecular determinants for the specificity of interactions between PGC-1α and the ERRs, whereby negative cooperativity prevails in the binding of the coactivators to these receptors. Our work indicates that allosteric regulation may be a general mechanism controlling the binding of the coactivators to homodimers.

## Introduction

**Figure 1:**
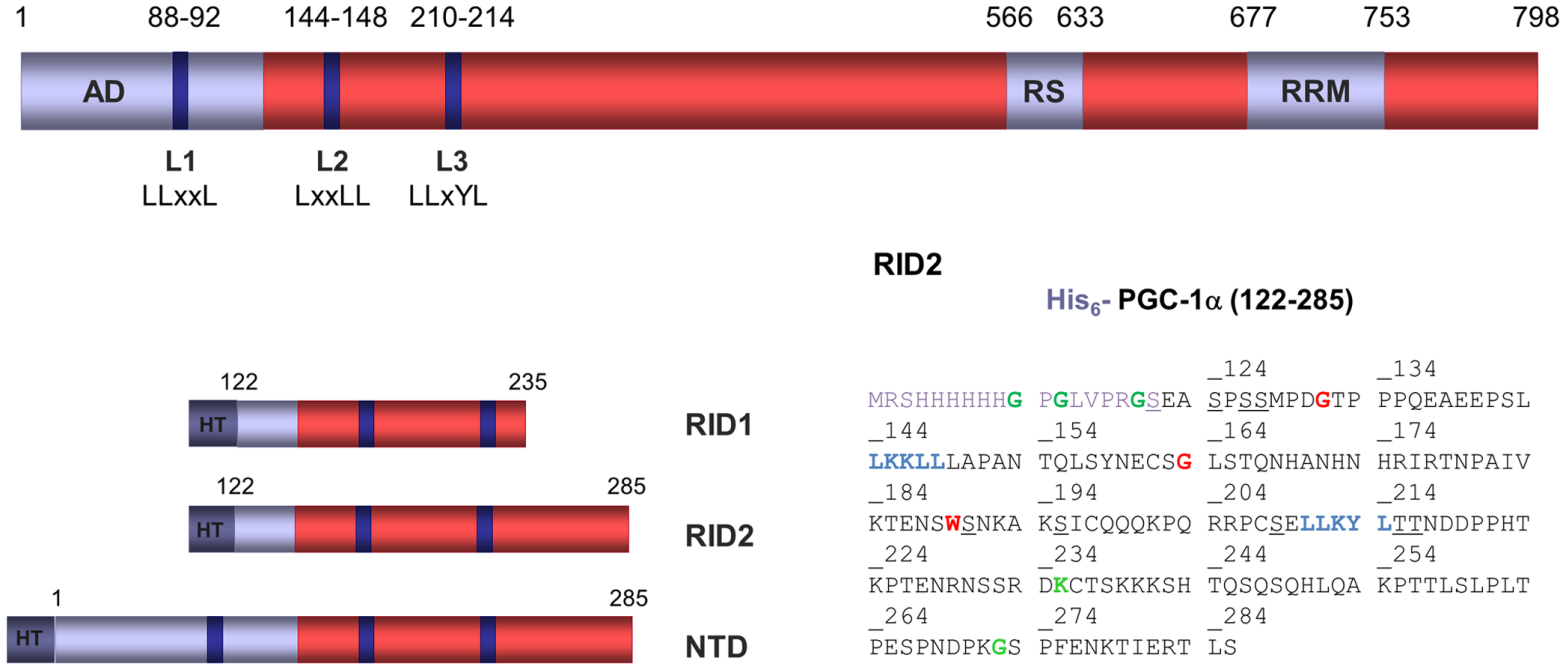
Architecture of human PGC-1α and constructs used in this study. PGC-1α contains three LxxLL motifs, two of which (L2 and L3) are responsible for specific interaction with NRs. The protein constructs used here are PGC-1α RID1 (122–235), PGC-1α RID2 (122–285) and PGC-1α NTD (1–285) that contain motifs L2 and L3. On the right, the sequence of the His_6_-tagged PGC-1α RID2 is given showing residues of the L2 and L3 motifs (bold, blue). Residues identified by NMR as being outside the interacting region are shown in green; residues unambiguously identified as being in the interacting region are shown in red; additional candidate Ser/Thr residues, affected by binding are underlined.

Gene transcription is a highly regulated and dynamic process orchestrated by large multiprotein complexes harboring different enzymatic activities. The regulation of gene expression is carried out by transcription factors (TFs), such as the nuclear receptors (NRs), the transcriptional activities of which are modulated by coactivators. In vertebrates, the regulation of cellular metabolism and energy homeostasis is strongly linked to peroxisome-proliferator-activated-receptor γ coactivator 1α (PGC-1α). PGC-1α has been implicated in numerous pathogenic conditions, including diabetes, obesity, neurodegeneration, cardiomyopathy and ischaemic diseases [1]–[8]. It has a unique ability to transduce a wide array of external physiological stimuli such as exposure to cold, fasting and physical exercise into transcriptional responses. Notably, PGC-1α induces and coordinates the expression of genes that regulate mitochondrial biogenesis, respiration and glucose homeostasis through coactivation of nuclear TFs, including the estrogen-related receptors ERRα and ERRγ, orphan NRs that interact directly with PGC-1α[9]–[12]. PGC-1αcontains a distinct set of domains, including a transcriptional activation domain and the major NR interacting domain (RID) in the N-terminal portion and an arginine-serine-rich domain and a RNA binding motif in the C-terminal portion [3] (**Fig. 1**). The interaction of PGC-1α with NRs occurs through leucine-rich interacting motifs which represent anchoring helices that have the potential to bind to a hydrophobic groove at the LBD surface [13]–[15]. Of three such motifs (L1, L2 and L3) found at the N-terminus of PGC-1α, only L2 and L3 were demonstrated to interact with NRs. The classical LxxLL L2 motif was shown to be the major binding site of NRs such as PPAR, ER, RXR and HNF4 [16]–[21]. On the other hand, the motif L3 of sequence LLxYL was initially suggested to function as the primary site of interaction of PGC-1α with ERRα and ERRγ [10], [11], [22]–[24], while motif L2 was shown to participate to a lesser extent to the interactions with ERR [10], [24]. In contrast to all reported data, the work of Devarakonda *et al* recently suggested that the binding of PGC-1α to ERRγ LBD is specific and different from that to ERRα and to other NRs, with motif L2, and not motif L3, being the major contributor to the interaction with ERRγ [25].

Here, we report the first comparative biophysical and structural study of the binding of PGC-1α RID to ERRα and to ERRγ. We show that PGC-1α RID binds in an identical manner to ERRα and ERRγ homodimers. Microscale thermophoresis (MST) [26] experiments provided us unambiguous experimental evidence demonstrating that the binding of PGC-1α RID is restricted to one subunit of the ERR homodimer only and involves the high affinity L3 and the low affinity L2 motifs. Apparent binding affinities measured by MST for wild-type and mutant PGC-1α RIDs are in remarkable agreement with values measured by isothermal titration calorimetry (ITC). NMR studies defined the limits of PGC-1α RID that interacts with ERR LBD. Consistent with biophysical data, small-angle X-ray scattering (SAXS) analysis demonstrates that the PGC-1α/ERRα and PGC-1α/ERRγ complexes have an identical architecture with an asymmetric binding of PGC-1α RID to one subunit of the receptor homodimer. The topology of the complex is ERR specific, as further emphasized by the comparison with data obtained for the PGC-1α RID bound PPARγ/RXRα LBD heterodimer. The molecular model gives insight into the specificity of the interaction between PGC-1α and the ERRs and helps understand how this versatile protein discriminates between ERRs and other NRs.

## Results

### The Partial Folding of PGC-1α RID upon Binding to ERRα and ERRγ Involves the Same Interacting Region

Several constructs of PGC-1α were designed to encompass the RID within different boundaries that encompassed motifs L2 and L3 (RID1, RID2 and NTD in **Fig. 1**). While sequence alignment of PGC-1α NTD indicates a high level of sequence conservation (**Fig. S1**) the analysis of the amino acid composition and disorder predictors suggests that PGC-1α NTD can be considered as an intrinsically disordered protein (IDP), while also retaining some features of ordered proteins (**Fig. S2**). The solution properties of the isolated RID1, RID2 and NTD PGC-1α constructs were examined experimentally using a range of biophysical characterization methods (**Fig. S3 and Table S1**). The data suggest that the PGC-1α RID fragments are less compact and more flexible than globular proteins. Consistent with this, the SAXS analysis (**see File S1**) showed that the structural parameters (*R*g and *D*max) of PGC-1α RID1, RID2 and NTD are larger than typical values estimated for globular proteins and even larger than values expected for IDPs, suggesting that the PGC-1α RID constructs are stiffer and thus more extended than classical IDPs (**Fig. S3 and Table S3**).

**Figure 2:**
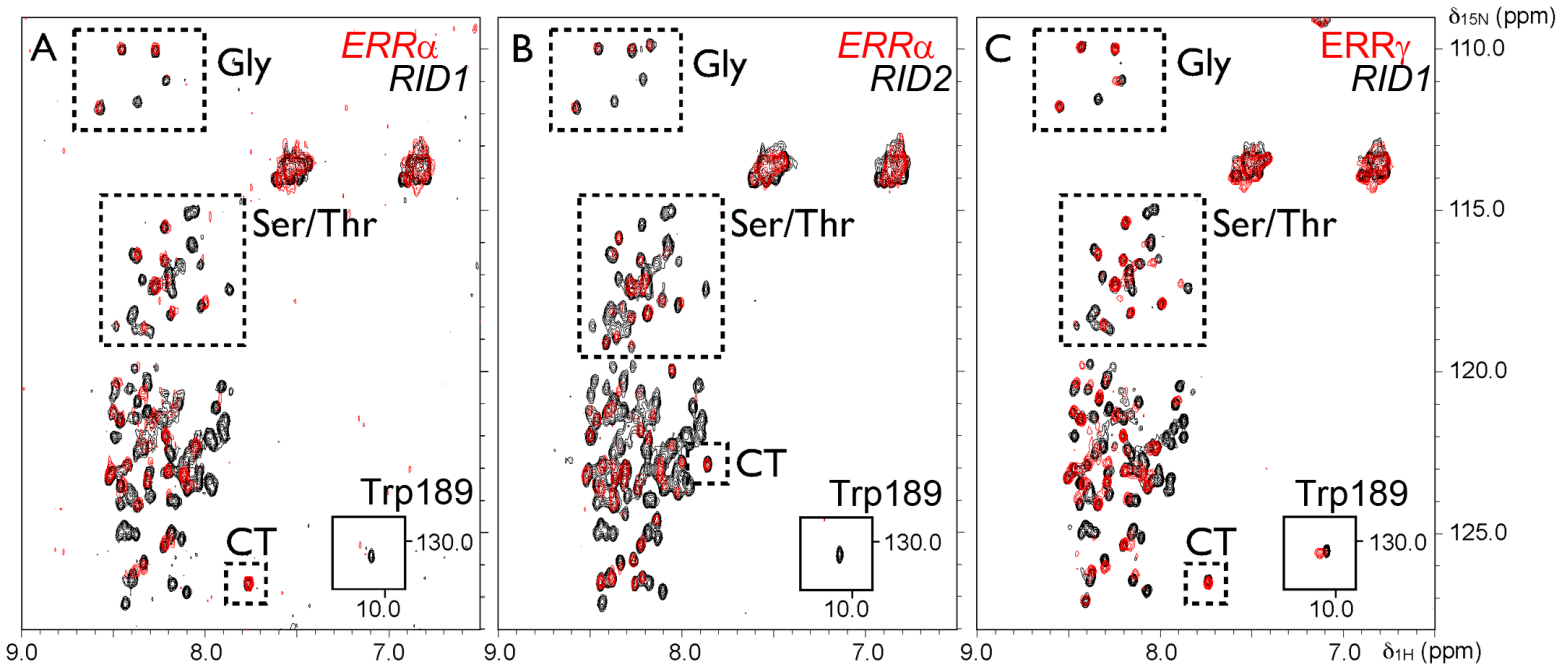
PGC-1α RID interacts through an identical region with ERRα and ERRγ LBD. NMR spectra of PGC-1α RID fragments in interaction with (**A–B**) ERRα LBD and (**C**) ERRγ LBD. ^1^H-^15^N HSQC spectra of PGC-1α RID1 (**A and C**) and RID2 (**B**) alone (black) and following addition of unlabeled ERR LBD (red). The sets of attenuated cross-peaks are broadly similar. The additional peaks in RID2 are not affected by binding to either ERR LBD. Cross-peaks in regions expected for Gly and Ser/Thr residues are boxed and the cross-peak that probably arises from the C-ter residue is indicated (CT). The cross-peak corresponding to W189 is boxed, showing the broadening or shift upon complex formation.

Given the intrinsic disorder properties of PGC-1α NMR was employed to gain insight into the conformational changes that PGC-1α RID undergoes upon interaction with ERRα and ERRγ LBD. The measurements on the isolated PGC-1α RID1 and RID2 indicate that the PGC-1α RID domains are essentially unstructured, in agreement with the computational and experimental analysis (**Figs. S3G-I)**. On interaction of ^15^N-labelled PGC-1α RID1 with either unlabeled ERR LBD, a set of more than 30 cross-peaks remain. For some portions of PGC-1α RID1, complex formation leads to line broadening and cross-peaks are lost from the spectrum. Other portions of the sequence remain flexible, so line-widths remain narrow and cross-peaks are retained (**Fig. 2** **)**. A small number of cross-peaks that are lost upon interaction with ERRα LBD are shifted upon interaction with ERRγ LBD. This may arise from differences in experimental conditions or a small intrinsic difference in exchange rates for the two complexes. The spectra allow us to deduce which portions of the sequences are intimately involved in complex formation and which remain unstructured. For this purpose, we did a comparative examination of the cross-peaks that remain, get attenuated or disappear in the spectra of the bound versus free PGC-1α RID1 and PGC-1α RID2 in interaction with ERRα and ERRγ LBD. We paid special attention to the cross-peaks belonging to glycine, tryptophan and threonine/serine residues, since these latter were clearly and unambiguously identified in the spectra (**see File S1**). The analysis suggests that binding of PGC-1α involves the polypeptide chain encompassing G131, G163 and W189 and that the interaction domain extends as far as T216, while it does not involve the N-terminal tag or the C-terminal residues. Importantly, the NMR data show that the same region of PGC-1α RID is involved in the interaction with either ERRα or ERRγ LBD.

### Only One PGC-1α RID Molecule is Bound to the ERRα and ERRγ LBD Homodimer

**Figure 3:**
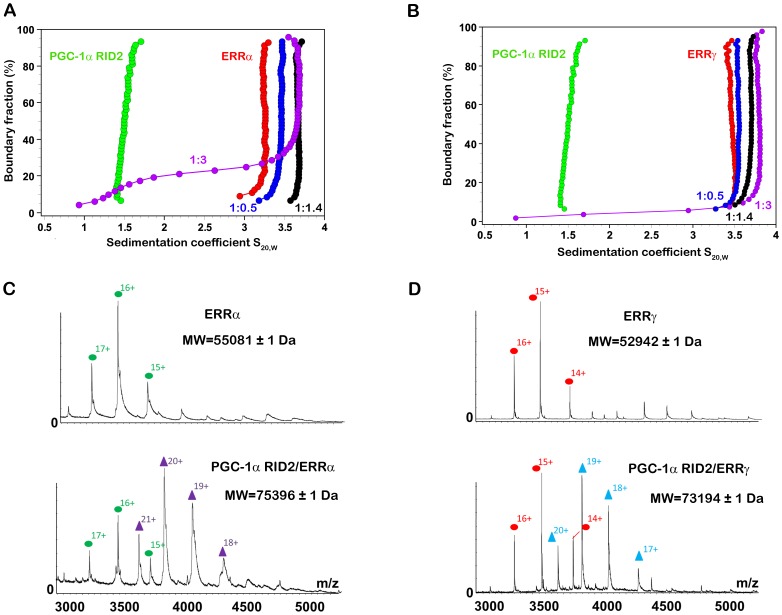
Biophysical characterization of the stoichiometry of the PGC-1α RID2/ERR complexes. **A** and **B**. SV-AUC experiments for a titration series of increasing molar ratio of PGC-1α RID2 with respect to (**A)** ERRα and (**B**) ERRγ LBD dimer. G(S_20,w_) distributions are shown over the entire boundary for free PGC-1α RID2 (green), free ERR LBD (red) and for the titration series with ERR LBD:PGC-1α RID2 ratios 1∶0.5 (blue), 1∶1.4 (black) and 1∶3 (magenta). The excess of PGC-1α RID2 in the 1∶3 data is seen as a shoulder extending to values close to that of free PGC-1α RID2. In the experiments with ERRγ LBD, the concentration of PGC-1α RID2 was overestimated, as can be seen by the faint shoulder of the 1∶3 titration data. Thus the 1∶1.4 molar ratio is overestimated and as a consequence the corresponding data approach a limiting value of saturation given by the 1∶3 titration data. **C** and **D**. ESI mass spectra recorded under non-denaturing conditions in 200 mM ammonium acetate at pH = 7.4 for (**C**) ERRα LBD (top) and PGC-1α RID2/ERRα (bottom) and for (**D**) ERRγ LBD (top) and PGC-1α RID2/ERRγ complex (bottom). The different charge states of the proteins are indicated above the peaks and depicted for ERRα and ERRγ with green and red dots, respectively and for PGC-1α RID2/ERRα and PGC-1α RID2/ERRγ with magenta and blue triangles, respectively. For the measurements of ERRα complexes, the His_6_-tag of ERRα LBD was not cleaved, resulting in an increase of 3766 Da with respect to the molecular weight shown in Table S1.

The stoichiometry of the PGC-1α/ERR complexes for both ERRα and ERRγ was determined using analytical ultracentrifugation in sedimentation velocity mode (SV-AUC). Formation of the complex was also followed by Tris/CAPS native PAGE electrophoretic mobility (**Fig. S4A**). Titration studies were carried out with increasing molar ratios of PGC-1α RID2 relative to the ERR LBD homodimer (0.5, 1.4 and 3 molar ratios relative to ERR dimer concentration). The titration series was analyzed in terms of diffusion-corrected integral distributions G(S_20,w_) of the sedimentation coefficients S_20,w_, using van Holde-Weischet plots (**Figs. 3A–3B** **and Table S1)** and by a two-dimensional spectrum analysis (2DSA) coupled to statistical genetic algorithm Monte-Carlo (GA-MC), where the sedimentation coefficients are reported together with the frictional ratios in a two-dimensional graph including statistical confidence intervals (**Figs. S4B and S4C**). The analysis of the titration series indicate that the S_20,w_ value of ERR LBD gets larger upon addition of 0.5 molar equivalents of PGC-1α RID2, further increases for the ERR complex with 1.4 molar equivalents of PGC-1α RID2, and stay unchanged for larger molar ratios. This is further supported by results from electrospray mass-spectrometry (ESI-MS) analysis performed under non-denaturing conditions [27], [28] **,** indicating that the PGC-1α RID2/ERR complex consists of one PGC-1α RID2 molecule per ERR homodimer (**Figs. 3C and 3D**). Finally, SEC-MALS analysis on PGC-1α RID2/ERRγ LBD led to the same conclusion (**Fig. S4D and Table S1 for the calculated molecular weights**). Altogether, our biophysical studies strongly indicate that only one PGC-1α RID molecule binds to the homodimeric ERR LBDs and that the binding of a second molecule is precluded at physiological concentrations.

### A Single ERR Receptor Subunit is Involved in the Binding of PGC-1α RID

**Figure 4:**
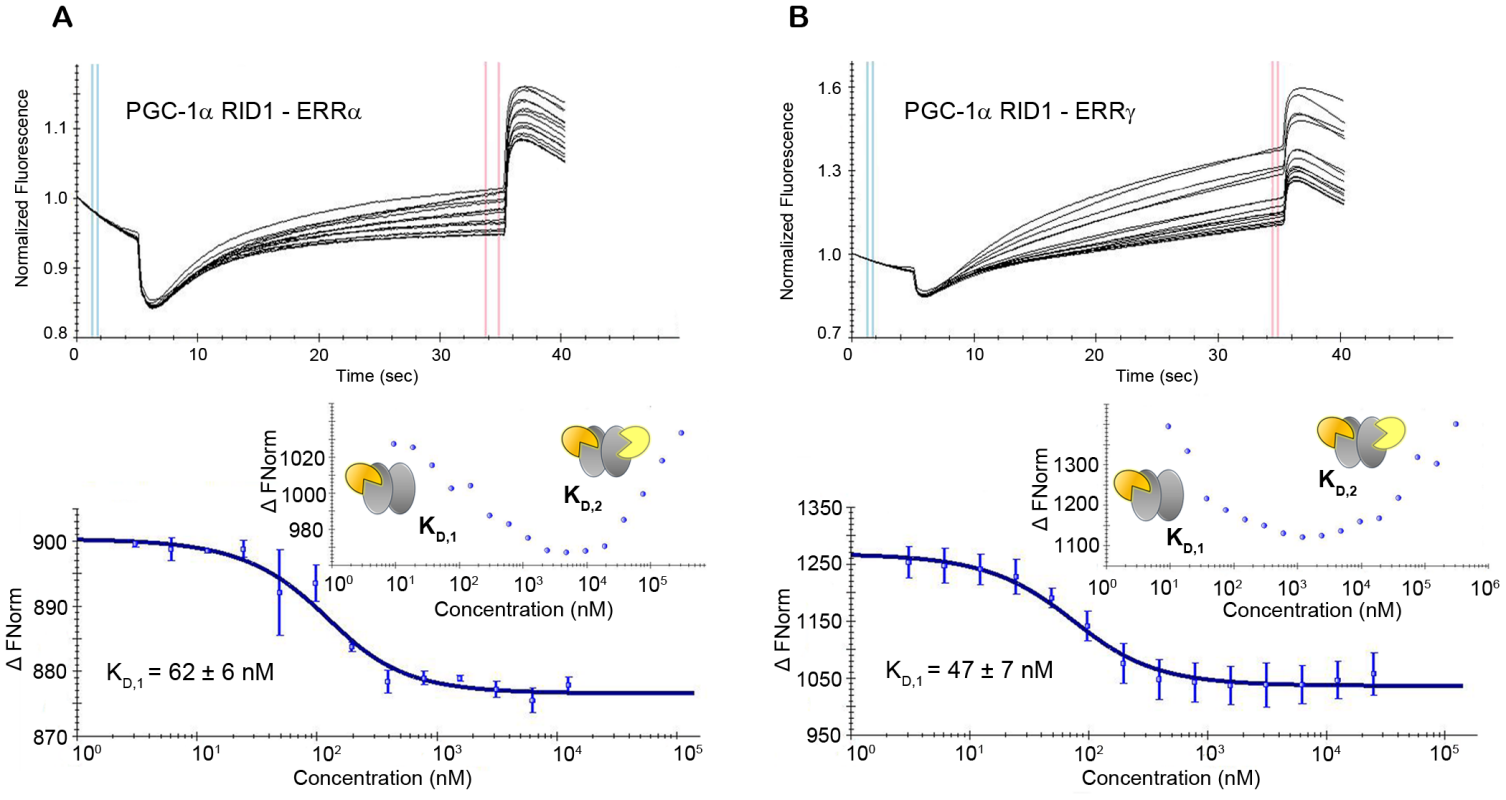
PGC-1α RID1 binding to ERRα and ERRγ measured by MST. Unlabeled PGC-1α RID1 protein was titrated into a fixed concentration of (**A**) labeled ERRα LBD and (**B**) labeled ERRγ LBD. Top panels: raw thermophoresis data recorded at 20°C using the LED at 50% and IR-laser at 80%. Bottom panels: isotherms averaged over three consecutive measurements and fitted according to the law of mass action to yield the apparent K_D,1_. For the determination of K_D,1_, the concentration of unlabeled PGC-1α RID1 was varied between 30 µM and 3 nM, while the concentration of ERR LBD was kept fixed (50 nM). Insets: isotherms for titration series comprising higher unlabeled PGC-1α RID1 concentrations (300 µM to 10 nM) with a fixed ERR LBD concentration (20 nM), showing the two binding events of binding affinities K_D,1_ and K_D,2_.

Knowing the stoichiometry, we thus asked the question whether PGC-1α RID is bound to ERRα and ERRγ through interactions with one subunit or with both subunits. In the former case, it is thought that the PGC-1α motif L3 is anchored in the canonical coactivator groove at the surface of the LBD, whereas motif L2 adopts a helical conformation and might form additional contacts with another part of the surface of the same LBD subunit. In the latter case, the binding of PGC-1α RID with the two subunits of the LBD dimer is supposed to involve the motifs L2 and L3, each interacting with the canonical coactivator groove of either receptor subunits, as proposed recently for PGC-1α/ERRγ LBD [25]. To discriminate between the binding of PGC-1α RID asymmetrically on one receptor subunit or both subunits in a sort of cap model, we employed microscale thermophoresis (MST), a novel, powerful method for the characterization of protein-protein; protein-DNA or RNA and protein-ligand interactions [26]. Using a titration approach, MST allows the affinity constants of interactions to be determined in the binding equilibrium. In MST experiments, one of the binding partners is fluorescently labeled at a fixed concentration (a few tens of nM), while the concentration of the unlabeled partner is varied from a high concentration, much above the expected dissociation constant, down to sub-stoichiometric concentrations with respect to the labeled protein. For our experiments, we considered the PGC-1α RID1 wild-type and the LxxLL mutants L2m and L3m, where the L2 and L3 motifs were selectively disrupted by alanine point mutations of the leucine residues inside the motifs. The thermophoresis curves corresponding to the binding of wild-type PGC-1α RID1 to ERRα and ERRγ show a similar profile with two transitions characterized by a change (positive or negative) of the thermophoresis value. The two transitions correspond to two distinct binding events of apparent binding affinity constants *K*_D,1_ and *K*_D,2_ (**Fig. 4** **, inset**). The first binding event, observed in the low coactivator concentration range, represents the binding of one molecule of PGC-1α RID1 to the receptor dimer (**Fig. 4**). It features a binding affinity constant *K*_D,1_ of the order of a few tens of nM for both ERRα and ERRγ (**Table 1**). The second transition is observed at larger coactivator concentration and is characterized by a lower binding affinity constant *K*_D,2_ of the order of µM. This second thermophoresis jump represents the binding of a second PGC-1α RID1 molecules to the already formed PGC-1α RID1/LBD dimer that occurs at very large molar excess of coactivator corresponding to non-physiological concentrations. Disrupting motif L2 or motif L3 of PGC-1α RID1 by point mutations does not affect the features of the binding curve as observed for wild-type PGC-1α RID1. However, the value of the binding affinity constant *K*_D,1_ of these mutants changes with respect to that of wild-type PGC-1α RID1 (**Table 1**). In fact, ERRα and ERRγ bind PGC-1α RID1 L2m (only L3 motif is functional) with a slightly lower binding affinity than the corresponding value for wild-type PGC-1α RID1. Mutating the L3 motif results in a much larger decrease in the binding affinity for ERRα and ERRγ, suggesting that motif L3 is the principal interaction motif. However, this also indicates that motif L2 contributes to the high-affinity binding of PGC-1α RID1 to ERRs, as demonstarted by the lack of binding of the double mutant L2mL3m (**Table 1**). Remarkably, the apparent *K*_D,1_ values calculated from MST experiments compare very well with *K*_D_ values obtained independently by isothermal titration calorimetry (ITC) (**Table 1** **Table S2 and Fig. S5**). Since in our MST experiments, there are some technical limitations in going to very high coactivator concentration (mM range), we were not able to measure the full binding curve for the second binding event, in particular the plateau region in the upper coactivator concentration range. Therefore, we could not determine precisely the binding affinity constant *K*_D,2_, but could only suggest an approximate value based on the initial rise. When we compared the curves of the L2m mutant and wild-type PGC-1α RID1, we see no obvious differences in the *K*_D,2_ values. However, by comparing the L3m mutant with the wild-type PGC-1α RID1, we observe an increase in the *K*_D,2_ value for the wild-type which is roughly the same order of magnitude as the corresponding increase of *K*_D,1_ value observed for the first binding event. These observations suggest that the second PGC-1α RID1 molecule binds to the second subunit of ERR homodimer with the same interaction patterns as that observed for the first PGC-1α RID1 molecule, i.e. main specific interactions through the L3 motif and secondary interactions carried by the L2 motif on the same receptor subunit. Altogether, the MST data demonstrate that the specific binding of the PGC-1α RID1 to ERR at physiological molar ratios involves a single ERR homodimer subunit.

**Table 1:** Apparent binding affinities of PGC-1α/ERR LBD from MST and ITC.

		MST			ITC
ERR LBD	PGC-1α RID1		*K*_D,1_ (nM)	*K*_D,2_ (µM)	*K*_D_ (nM)
ERRα	Wild type		62±6	42	71±6
	L2m		119±9	45	115±7
	L3m		609±54	>290	369±66
	L2mL3m		No binding	No binding	No binding
ERRγ	Wild type		47±7	80	53±3
	L2m		91±6	84	71±6
	L3m		517±45	>284	223±27
	L2mL3m		No binding	No binding	No binding

ITC data determined at 20°C and at pH = 7.5 as described in *SI.*

### SAXS Provides Evidence for Similar Induced Partial Compaction of PGC-1α on Interaction with ERRα and ERRγ

**Figure 5:**
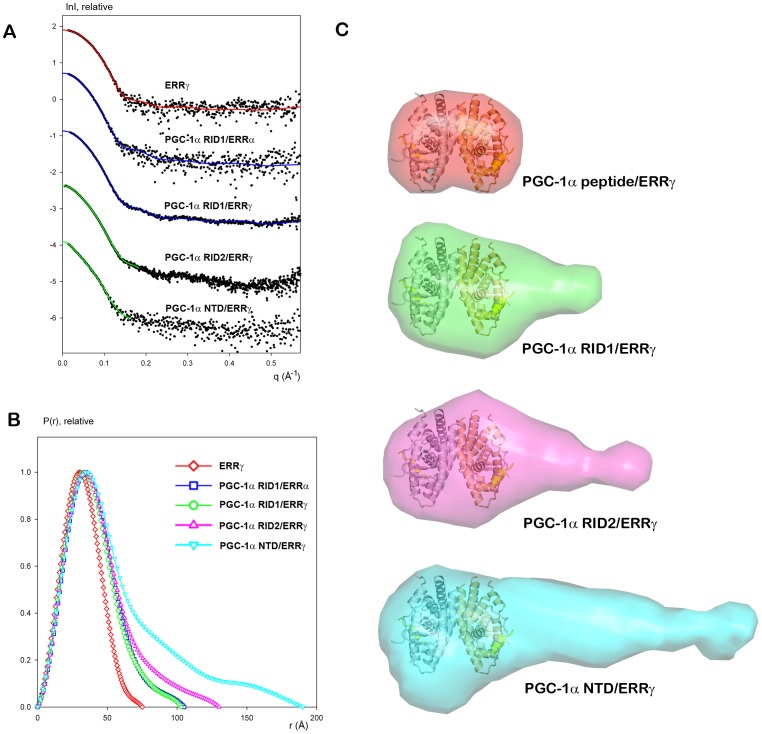
SAXS of ERR and ERR in complex with PGC-1α RID. **A**. Scattering profiles of (from top to bottom) ERRγ LBD, PGC-1α RID1/ERRα, PGC-1α RID1/ERRγ, PGC-1α RID2/ERRγ and PGC-1α NTD/ERRγ. Experimental data are denoted by black dots, the corresponding fits are given as solid lines. Red fit is computed from the crystal structure of ERRγ LBD (PDB entry 1KV6). Blue fits are yielded by the best MD based model of PGC-1α RID1/ERRγ. Green lines represent the fits to the experimental data by the appropriate *ab initio* bead models. The profiles are arbitrary displaced in logarithmic scale for better visualization. **B**. Distance distribution functions of ERRγ LBD (red), PGC-1α RID1/ERRα (blue) PGC-1α RID1/ERRγ (green), PGC-1α RID2/ERRγ (magenta) and PGC-1α NTD/ERRγ (cyan) computed from the X-ray scattering patterns using the program GNOM. **C**. *Ab initio* molecular envelopes from SAXS measurements. The *ab initio* molecular envelope of ERRγ LBD alone is shown in semi-transparent red color together with the crystal structure of ERRγ LBD bound to short peptides derived from the coactivator SCR-1 (PDB code 1KV6). The *ab initio* molecular envelopes of the complexes between ERRγ LBD and PGC-1α RID1 (green), PGC-1α RID2 (magenta) and PGC-1α NTD (cyan) are depicted highlighting the location of the additional 50 amino acids at the C-terminal of the PGC-1α RID2 and of the N-terminal extension of PGC-1α NTD. The Cα-trace of ERRγ LBD homodimer and of the coactivator peptides are shown in grey and yellow ribbons, respectively.

As references for PGC-1α/ERR complexes, scattering data were first obtained for the isolated ERRα and ERRγ LBDs. Guinier analysis showed a linear behavior for both LBDs with no sign of unspecific aggregation and allowed the determination of R_g_ (**Table 2**). The experimental SAXS data for ERRγ is neatly fitted by the scattering profile calculated from the crystal structure (PDB entry 1KV6) using CRYSOL [29] (**Fig. 5A**) and yields a symmetrical distance distribution function (**Fig.5B**). As expected, the Kratky plot corresponds to a folded globular protein (**Fig. S3F**). For ERRα LBD, we have detected a minor fraction of specific multimerization of the ERR dimers (trimers of dimers). These hexamers were found to occur in the crystal packing of ERRα LBD (PDB code: 2PJL) and exist as a minor fraction of the molecules in solution, as estimated by the program OLIGOMER [30]. The latter gives an estimate of 0.2±0.02 volume fraction of hexamers compared to dimers (PDB code: 3D24), resulting in a reasonable fit with a chi value of 1.07. The scattering intensities were then recorded for several PGC-1α RID/ERR complexes (**Fig. 5A**) and the structural parameters were derived from the data (**Fig. 5B** **and** **Table 2**). The R_g_ and D_max_ values of the PGC-1α RID complexes are significantly smaller than the corresponding value of the isolated PGC-1α RID molecules (**Table 2** **and** **Table S3**). This suggests that PGC-1α RID undergoes a partial compaction upon binding to its ERR partner and the similarity in the values of the structural parameters indicates a similar type of compaction upon complex formation. The partial induced folding is dependent on the presence of the two interaction motifs, as suggested by the much larger parameters observed for the PGC-1α RID1 L2m complex, which are comparable to the values measured for PGC-1α RID1/PPAR-RXR (**Table 2**). For PGC-1α RID2/ERRγ LBD, the increase in R_g_ and D_max_, compared to those of PGC-1α RID1/ERRγ is large considering for an extension of 50 amino acids. This is in agreement with the NMR data that showed that this additional region (PGC-1α 236–285) at the C-terminus is disordered and does not interact with ERR.

**Table 2:** Structural parameters of ERRα, ERRγ and their complexes with PGC-1α RIDs from SAXS analysis.

Sample	R_g_ (Å)*	D_max_ (Å) #,&	Type of measurement$	Sample concentration(mg/mL)
ERRα LBD	25.6±0.6	80±5	SEC	19.0
	25.6±0.6	n.d.	Direct	1.9
ERRγ LBD	24.5±0.5	n.d.	Direct	1.25
	24.4±0.5	n.d.	Direct	1.6
	24.5±0.5	75±5	Direct	2.5
	24.9±0.6	n.d.	Direct	5.0
PGC-1α RID1/ERRα LBD	29.4±0.7	105±5	Direct	1.8
	30.3±0.7	n.d.	Direct	2.2
	30.1±0.7	n.d.	Direct	11.0
PGC-1α RID1/ERRγ LBD	29.3±0.7	103±5	Direct	1.5
	30.1±0.5	n.d.	Direct	7.5
PGC-1α RID1(L2mut)/ERRγ LBD	39.7±0.7	160±10	Direct	1.8
	41.7±0.7	n.d.	Direct	3.7
PGC-1α RID2/ERRγ LBD	32.7±0.7	n.d.	Direct	1.1
	33.1±0.5	n.d.	Direct	1.2
	33.3±0.7	135±7	Direct	4.5
PGC-1α NTD/ERRγ LBD	44.0±1.5	190±10	SEC	8.2
PPARγ/RXR LBD	27.2±0.5	85±5	Direct	2.7
PGC-1α RID1/PPARγ/RXR LBD	36.0±1.0	165±10	Direct	4.0

* From Guinier analysis.

# From GNOM analysis.

& n.d. non determined.

$ Size-exclusion chromatography(SEC) or Direct injection into SAXS cell (Direct).

The solution structures of PGC-1α on interaction with ERRα and ERRγ show a highly similar asymmetrical topology.

The scattering curves of PGC-1α RID1/ERRα and PGC-1α RID1/ERRγ are very similar with almost identical R_g_ and D_max_ values and distribution functions P(r) (**Figs. 5A and B**). This strongly suggests that the architecture of the two complexes is alike with the same molecular determinants of complex formation. The partial multimerization of ERRα dimers observed in the free state is suppressed by binding of PGC-1α RID1, as suggested by the similar structural parameters of the two isotypes of ERR LBD in complex with PGC-1α RID1. This can be explained by the fact that PGC-1a is bound close to the hexamerization interface seen in the crystal packing of isolated ERRα LBD (PDB code: 2PJL). The molecular envelopes of the ERRα and ERRγ in complex with PGC-1α RID1 exhibit highly similar shape and size with a marked asymmetry as compared to the crystal structure of ERRγ LBD dimer (**Figs. 5C** **and** **6C**). From the shape of the molecular envelopes, it is tempting to position the LBDs in the globular region and the PGC-1α RID1 in the asymmetric tail of the envelope. The envelope of PGC-1α RID2/ERRγ shows an even more pronounced asymmetry as compared to that of PGC-1α RID1/ERRγ (**Fig. 5C**). This supports the positioning of PGC-1α in the asymmetric tail of the envelope where the extra portion arises from the 50 additional disordered residues at the C-ter of PGC-1α RID2. Similarly, the molecular envelope of PGC-1α NTD/ERRγ shows features resembling the envelopes of the complexes with the shorter PGC-1α RIDs, but with a longer tail (**Fig. 5C**). We can thus infer that the N-terminal extension is positioned asymmetrically on the side of the interacting ERR subunit.

**Figure 6:**
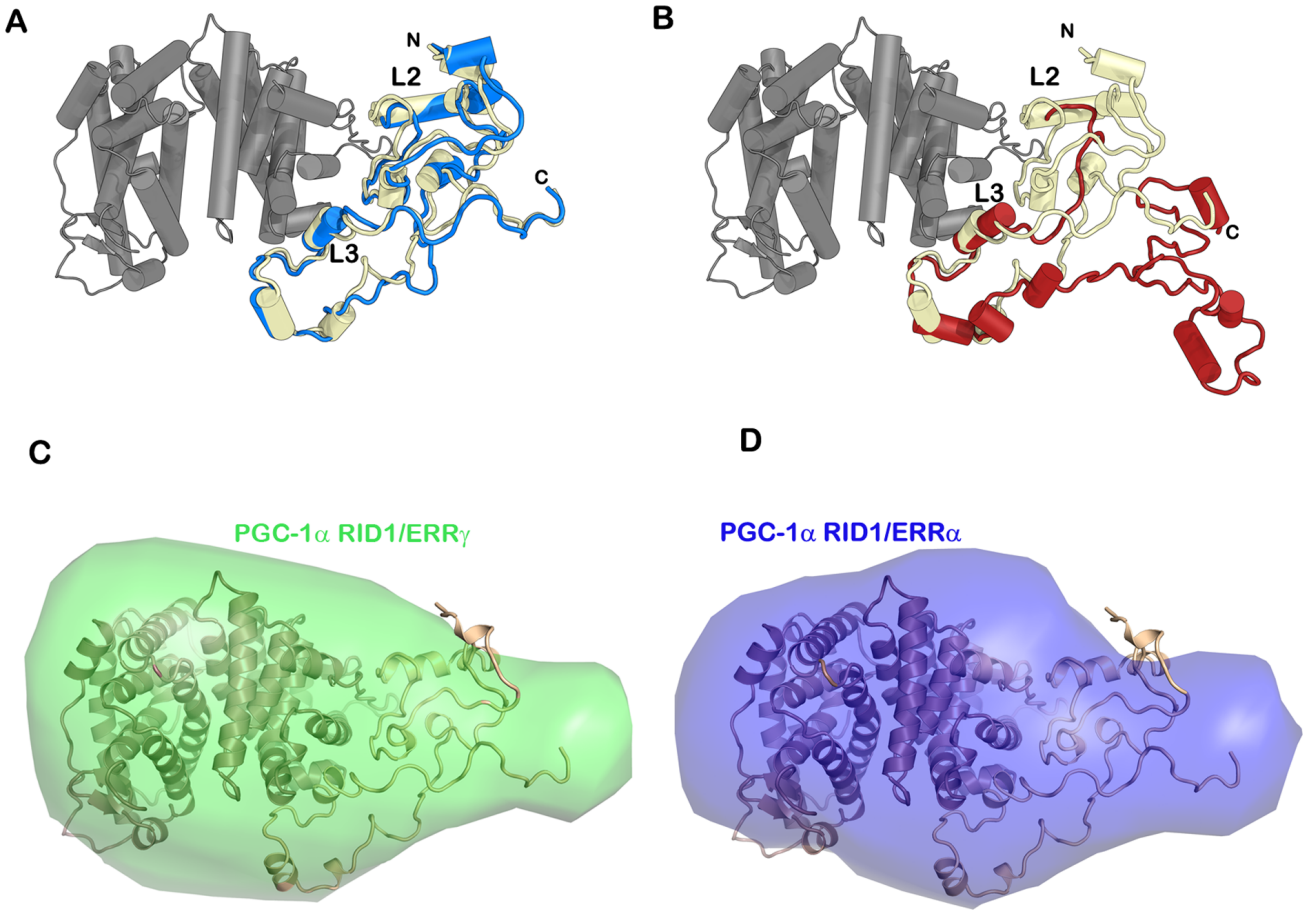
Solution structure of PGC-1α RID1/ERRγ and comparison with the *ab initio* envelope of PGC-1α RID1/ERRα. **A**. and **B**. Schematic cartoon representations of the pseudo-atomic solution structures of the complex PGC-1α RID1/ERRγ. The Cα-trace of the PGC-1α RID1 molecule resulting from the rigid-body refinement is shown in light yellow. The N- and C-terminal ends and the positions of motifs L2 and L3, both of which corresponding to helices, are indicated. The Cα-trace of ERRγ LBD dimer is shown in dark grey (PDB entry 1KV6). **A**. One of the representative model obtained after relaxation using MD at moderate temperature is shown in blue. **B**. One of the representative model obtained along the trajectory of the higher temperature MD run is depicted in red. **C** and **D**. Molecular envelopes of the complexes PGC-1α RID1/ERRγ (green) and PGC-1α RID1/ERRα (blue) together with the pseudo-atomic solution structure of PGC-1α RID1/ERRγ shown in (A). The orientation of the models in C and D is identical to that in A and B.

Although SAXS is not a high resolution structural method, it provides valuable information on the topological arrangement of the various subunits composing a molecular complex [31]. When high-resolution structures of the subunits are available, detailed models can be constructed by rigid-body modeling, thus giving insight into the quaternary structure of the complex. Domains or subunits devoid of high-resolution structure can be either built by homology modeling or the secondary structure can be predicted de novo. In the case of PGC-1α RID1/ERRγ, the crystal structure of ERRγ LBD is available, whereas no structural data exists for PGC-1α. We therefore relied on ROBETTA [32], a powerful full-chain protein structure prediction server based on Rosetta comparative and ab initio modeling methods, to build model structures for PGC-1α RID1. A series of models was predicted for PGC-1α RID1 that all share similar features (**Fig.S6**). In particular, the structure of PGC-1α RID1 is predicted to be mainly composed of loops interspersed with short helical stretches which notably encompass motifs L2 and L3, in agreement with the analysis of amino acid composition and the disorder predictions. As a starting model for PGC-1α RID1/ERRγ, we used one of the Robetta models for PGC-1α RID1 and manually positioned it together with the ERRγ LBD structure in the molecular envelope obtained from experimental scattering data. For this purpose, motif L3 was anchored into the coactivator binding groove of ERRγ, as observed in crystal structures of ERRα bound to PGC-1α L3 peptides [33]–[35]. Given the observations made on the larger complexes, we placed the N- and C-termini away from the LBD, but on the same side of the receptor. As a result, motif L2 lies in close vicinity of the receptor body, though the resolution of SAXS data is not sufficient to assess the validity of this outcome. Next, rigid-body refinement against the scattering data was performed using SASREF [36] to adjust the position of PGC-1α RID1 with respect to the LBD using contacts restraints to keep motif L3 in the binding site. The resulting models agree pretty well with the experimental data, but with some mismatch in the q region close to 0.15 Å^-1^. In order to get the best SAXS compatible model of PGC-1α RID1/ERR LBD, we considered the model described above as an initial model for molecular dynamics (MD) simulations. The latter allow artifacts, such as unusual torsion angles and clashes between non-C_α_ atoms neglected in the course of restrained rigid body modeling, to be removed. MD calculations performed at moderate temperature allow the relaxation of the conformation of PGC-1α RID1, whereby positional constraints were imposed solely on the helical segment encompassing L3. As a result, several conformations were obtained which do not differ significantly from the starting model (**Fig. 6A**). In contrast, when the temperature of the MD trajectory is increased, a larger variety of conformations is obtained with significant deviation from the initial model (**Fig. 6B**). The scattering pattern for each MD atomic model was computed and compared to the experimental scattering data. Excellent agreement between the experimental and computed data is obtained for the atomic models equilibrated at moderate temperature, including around to q = 0.15 Å^-1^ (**Figs. 5A** **and** **6A** **and Table S4**). In contrast, clear mismatch between experimental and computed scattering intensities is seen for the series of atomic models sampled along the high temperature MD run, where the starting and the final MD models are shown in **Fig. 6B**. The MD analysis supports the validity of the model built for PGC-1α RID1/ERRγ, since even slight variations of the initial models result in computed scattering curves that deviate from the experimental curve. This result underlines the existence, in solution, of a well-defined compact conformation for PGC-1α RID1 bound to ERRγ. The validity of the model built and refined for PGC-1α RID1/ERRγ was examined in the case of PGC-1α RID1/ERRα (**Fig. 6D**), where the measured scattering curve agrees very well with the computed scattering curve for the PGC-1α RID1/ERRγ (**Fig. 5A** **and Table S4**), further supporting our initial observations.

## Discussion

In our structural and biophysical solution studies, we have gained considerable insight into the molecular mechanisms underlying the binding of PGC-1α to ERRα and ERRγ. The NMR analysis suggests that a minimal region of PGC-1α RID comprising residues G131 to T216 partly folds on interaction with ERR LBD. Furthermore, biophysical data consistently demonstrate that the binding of PGC-1α to ERR occurs with a stoichiometry of one PGC-1α molecule per ERR homodimer. Crucially, the combination of methods allowed us to demonstrate that the binding of PGC-1α RID to ERRα and ERRγ is identical and involve only one subunit of the ERR homodimer. The binding curves for the interaction of PGC-1α RID1 with ERRα or with ERRγ LBD were obtained independently by MST and ITC and suggest a specific and high affinity binding of one PGC-1α molecule to the receptor. Furthermore, the MST and ITC analysis strongly suggested that the principal interaction motif of PGC-1α RID with both receptors is motif L3, in agreement with all reported data [10], [11], [22]–[24], with the exception of the work of Devarakonda *et al* [25] who claim that the binding of PGC-1α to ERRγ LBD is specific and different from that to ERRα and to other nuclear receptors. These authors interpret hydrogen-deuterium exchange and ITC experiments as evidence that motif L2 is the main interaction motif of PGC-1α with ERRγ. We seriously question the validity of their data, since the PGC-1α construct (136–220) used in these measurements does not include the whole interaction region with ERRs as determined here by NMR and motif L2 lies on the extreme of the construct. The authors reported binding affinities for the binding of wild-type and mutant PGC-1α RID(136–220) to ERRγ LBD of the order of µM (Table S1 in [25]). These values are of the same order of magnitude as the values that we measured for the binding to ERR LBDs of short peptides encompassing either motif L2 (6.1 and 1.2 µM for ERRα and ERRγ, resp.) or L3 (4.1 and 1.3 µM for ERRα and ERRγ, resp.), but by far much higher than the values reported here, using two different methods. This is a strong indication that their PGC-1α RID (136–220) construct might be suboptimal for biophysical and structural studies.

The MST studies of the binding of PGC-1α to ERRs have allowed us to examine a range of concentration ratios between the two binding partners that cannot be reached with ITC. The resulting MST data demonstrate that the binding of a second PGC-1α RID molecule to the ERR homodimer is possible at huge concentrations of cofactor and with low binding affinity. Remarkably, the MST data provide critical and unique information on the topological arrangement of the PGC-1α RID/ERR LBD complexes, since they demonstrated that the high-affinity binding of PGC-1α to ERR involves only one ERR subunit. This contrasts with observations from crystallographic studies of ERR, ER and RAR LBD homodimers bound to short co-activator peptides, where two peptides are bound to each of the subunits of the homodimer [33]–[35], [37]–[39] (**Fig. 5C**). The presence of two peptides can be rationalized by the large molar excess used in the crystallization conditions. However, a careful analysis of these apparently symmetrical crystal structures reveals a difference in the mode of binding of the peptides to the homodimer, most likely reflecting different binding affinities between the two sites. Here, using PGC-1α RID rather than PGC-1α peptides, our data provided unambiguous evidence of the asymmetrical binding of PGC-1α to only one subunit of the ERR homodimers, implying that allosteric mechanisms prevent the binding of a second PGC-1α molecule to the ERR homodimer. This cannot be rationalized by steric hindrance, since the second binding site is located on the opposite side of the complex. In the case of the heterodimer RAR/RXR, it was recently shown that co-activator fragments bind preferentially to the NR partner of RXR and that binding of a second molecule to RXR is not favored [40]. However, in the case of heterodimers, the expected difference in binding affinity to the two different subunits is sufficient to explain the observed stoichiometry. The existence of allosteric control in the binding of ligands and/or coregulators to homodimeric receptors is much more intriguing, and has already been suggested for homodimeric RAR and ER [37], [39], whereby the binding of a ligand and/or a coregulator to one of the receptor subunit can impact on the binding of the ligand and/or a coregulator to the second subunit of the homodimer. Our data on PGC-1α RID/ERR LBD not only strongly support the hypothesis of allosteric regulation but demonstrate its existence. Thus, as a general consequence for both homo- and heterodimers, the binding of a coregulator to one subunit of the NR dimer enables further interactions with other regulatory proteins and may thus lead to fine-tuning of the transcriptional regulatory response by NRs.

Our scattering data suggest an identical architecture for ERRα and ERRγ complexes, with asymmetrical binding of PGC-1α to the receptor homodimer. By studying both ERRγ and ERRα in complex with three different constructs of PGC-1α RID, we are able to achieve unequivocal localization of the N- and C-terminal extensions of the RID and provide insight into the molecular mechanism of ERR recognition through motif L3, with motif L2 participating additively to the binding to ERR and contributing to the K_D,1 value_ (which is lowered by two-fold in the L2m mutant, see **Table 1**). Motif L2 may be necessary for stabilizing intra-molecular interactions allowing the compaction of the molecule upon interaction with ERR or for interactions with the receptor outside the canonical coactivator groove. Crystallographic studies of the complex would help solving this important issue. Our solution structures are consistent with the crystallographic observation that the N-terminal flanking residues of motif L3 contributes to the specificity of PGC-1α for ERRs through the formation of additional contacts with helix H4 and the connecting loop between helices H8 and H9 [33]. In contrast, the solution model proposed by Devarakonda *et al* of PGC-1α (1–220)/ERRγ LBD, in which L2 and L3 each interact with one subunit of the ERRγ homodimer, does not explain any of the binding specificity of PGC-1α to ERRα and ERRγ.

The peculiar binding mode of PGC-1α to one subunit of the ERRα and ERRγ homodimers represents a specific functional architecture which ought to be different from that obtained with other NRs, where the L2 motif of PGC-1α is the principal interaction motif [18]–[21]. A preliminary SAXS study of PGC-1α RID1 bound to PPARγ/RXR LBD supports this argument (**Table 2**); notably, the much larger increase in *R*_g_ and *D*_max_ of PGC-1α RID1/PPARγ/RXR as compared to the values for PGC-1α RID1/ERR suggests that PGC-1α RID1 does not fold in a compact manner upon interaction with PPARγ, through motif L2, but retains an extended conformation. This is radically different from the complexes with ERRs. As a result, the specific architecture of the PGC-1α/NR building block is expected to affect the mode of recruitment of other coactivator molecules to the transcriptional complex, with critical functional consequences. A question arises as to what extend the symmetry of the ERR homodimers is broken. PGC-1α may bind to the symmetric homodimer first, thus determining how the resulting asymmetric complex interacts with the non-symmetric DNA target. Alternatively, the prior binding of the ERR homodimer to the non-symmetric DNA target may provide a pre-existing asymmetric complex with a preferential side for the binding of PGC-1α. Further work is required to address this issue. Our studies provide the molecular determinants for the specificity of interactions and suggest that negative cooperativity is an essential mechanism controlling the binding of PGC-1α to ERRs. This work indicates that allosteric regulation may be a general mechanism controlling the binding of the coactivators to homodimers. Finally, because of the key role of PGC-1α/ERR in regulating energy homeostasis and its implication in metabolic diseases, the present study may provide clues for drug-design targeting the recognition interface [41].

## Materials and Methods

### Cloning, Protein Expression and Purification

ERRα-189-423, PGC1α RID1 (wild type and mutants), RID2 and NTD cloned in pET24b and ERRγ 229–458 cloned in pET15b were purified by affinity chromatography, followed by further polishing on gel filtration columns (GE healthcare).

### Nuclear Magnetic Resonance Experiments

^1^H-^15^N HSQC spectra were recorded at 600 MHz or 700 MHz and at 10°C on a Bruker DRX600 or Avance III 700 spectrometer equipped with a z-gradient triple-resonance cryoprobe. ^15^N-labelled protein samples were at a concentration of 100 µM. The molar excess of non-labelled ERR LBD partner was about 1.2 per PGC-1α RID molecule. The water signal was suppressed using the WATERGATE sequence [42]. Data were processed using NMRPipe [43] and analyzed with NEASY [44].

### Small Angle X-ray Scattering

Synchrotron X-ray solution scattering data were collected at the X33 beamline (DESY, Hamburg) and at the SWING beamline at SOLEIL Synchrotron (Gif-sur-Yvette, France). The data were processed and analyzed with the ATSAS suite of SAXS programs as described in *SI Text*. The figures of the envelopes and the solution structures were made using Pymol 1.4.1, Chimera 1.5.3 and the plots were drawn by using Sigmaplot 11.

### Microscale Thermophoresis

Experiments were performed using the Monolith NT 115 from NanoTemper Technologies GmbH and data were analyzed using NanoTemper Analysis software v.1.4.23. More details can be found in *SI Text.*

### Isothermal Titration Calorimetry (ITC)

Isothermal titration calorimetry experiments were performed using a MicroCal iTC200 (GE Healthcare) microcalorimeter. Data was analyzed with the software Origin 7.0 (OriginLab) using the one set of sites model. More details can be found in *SI Text.*

### Analytical Ultracentrifigation

Experiments were performed using a Beckman Proteomelab XL-I ultracentrifuge (Beckman Instruments, CA, USA) with an An50Ti 8-hole rotor. Data were fit using the UltraScan software version 9.9 as described in *SI Text*.

### Size-Exclusion Chromatography Coupled to Multi-Angle and Quasi-Elastic Light Scattering

SEC-MALS/QELS experiments were performed on a multi-angle light scattering detector (miniDAWN TREOS, Wyatt Technologies) coupled in-line with SEC and an interferometric refractometer (Optilab T-rEX, Wyatt Technologies). The analysis of the data was performed using the ASTRA 5.3.4 software (Wyatt Technologies).

### Electrospray Ionization Mass Spectrometry

ESI-MS measurements were performed on an ESI-TOF mass spectrometer (MicrOTOF, Bruker Daltonic, Germany). The non-denaturing mass measurements of the non-covalent complexes were performed in ammonium acetate (200 mM; pH 7.4) as described in *SI Text*.

## Supporting Information

Figure S1 (TIF)

Figure S2 (TIF)

Figure S3 (TIF)

Figure S4 (TIF)

Figure S5 (TIF)

Figure S6 (TIF)

Table S1 (DOCX)

Table S2 (DOCX)

Table S3 (DOCX)

Table S4 (DOCX)

File S1 (DOCX)
