# Supplementary figures and images for: The Asymmetric Binding of PGC-1α to the ERRα and ERRγ Nuclear Receptor Homodimers Involves a Similar Recognition Mechanism
Source: PLoS One. 2013 Jul 9;8(7):e67810. doi: 10.1371/journal.pone.0067810 (PMC3706463; doi:10.1371/journal.pone.0067810)

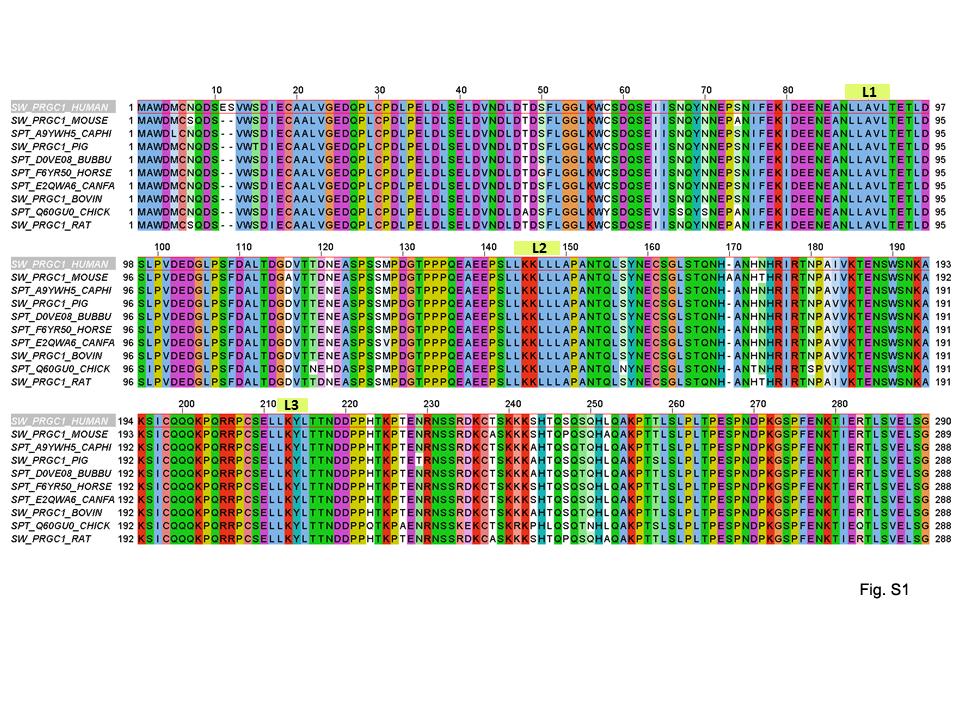

Supplement: Figure S1 — (TIF) [file pone.0067810.s001.tif]

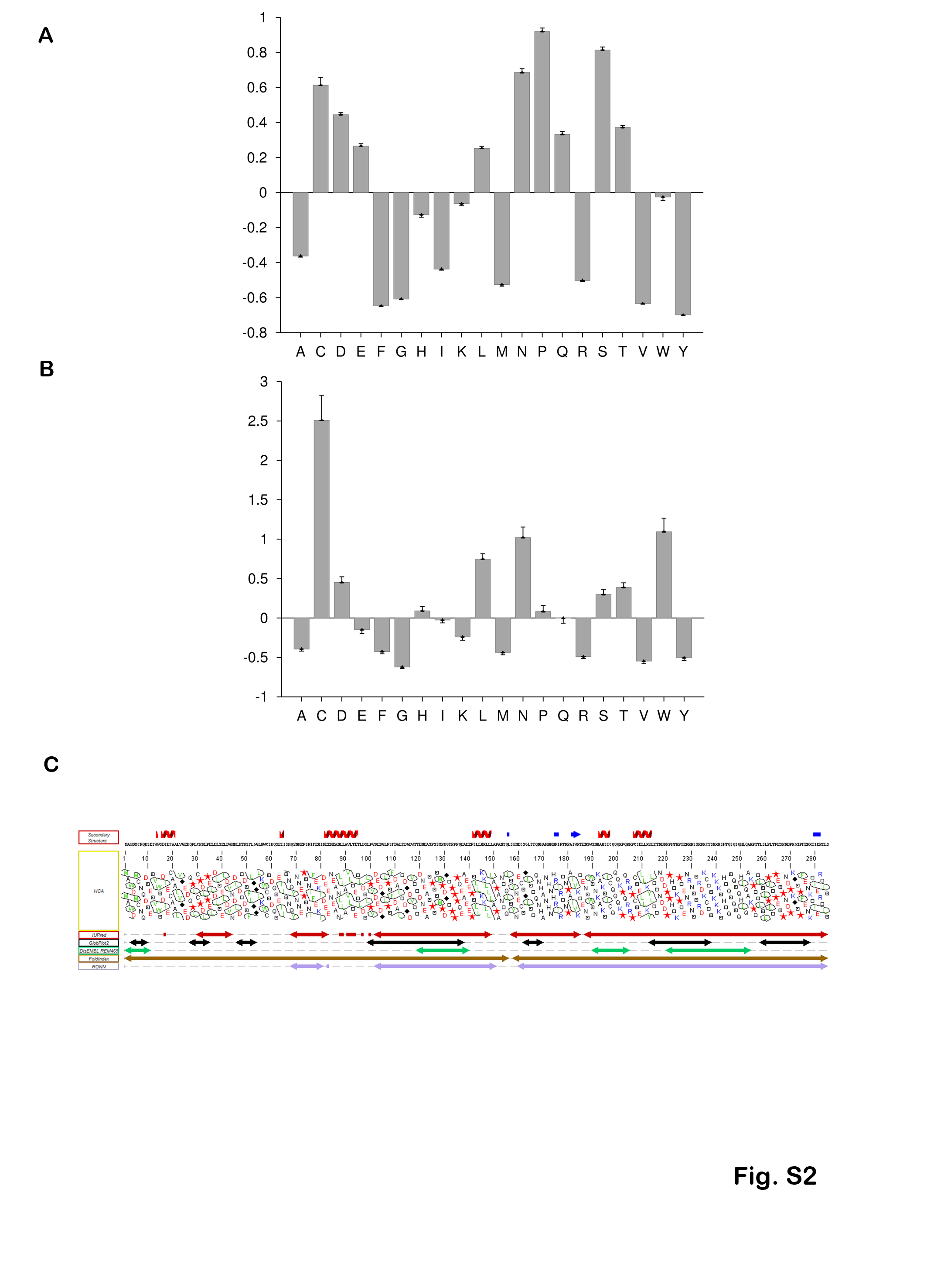

Supplement: Figure S2 — (TIF) [file pone.0067810.s002.tif]

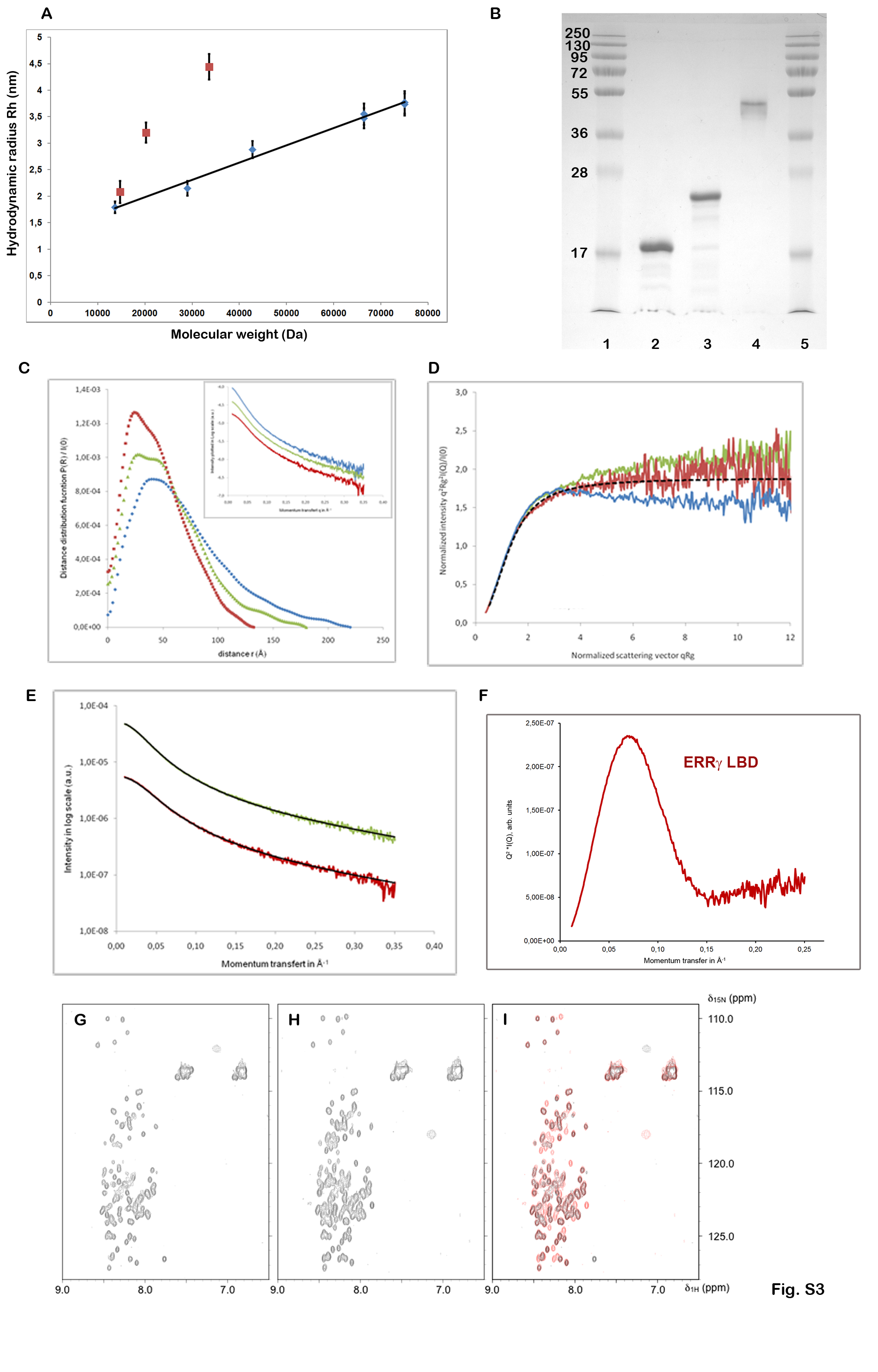

Supplement: Figure S3 — (TIF) [file pone.0067810.s003.tif]

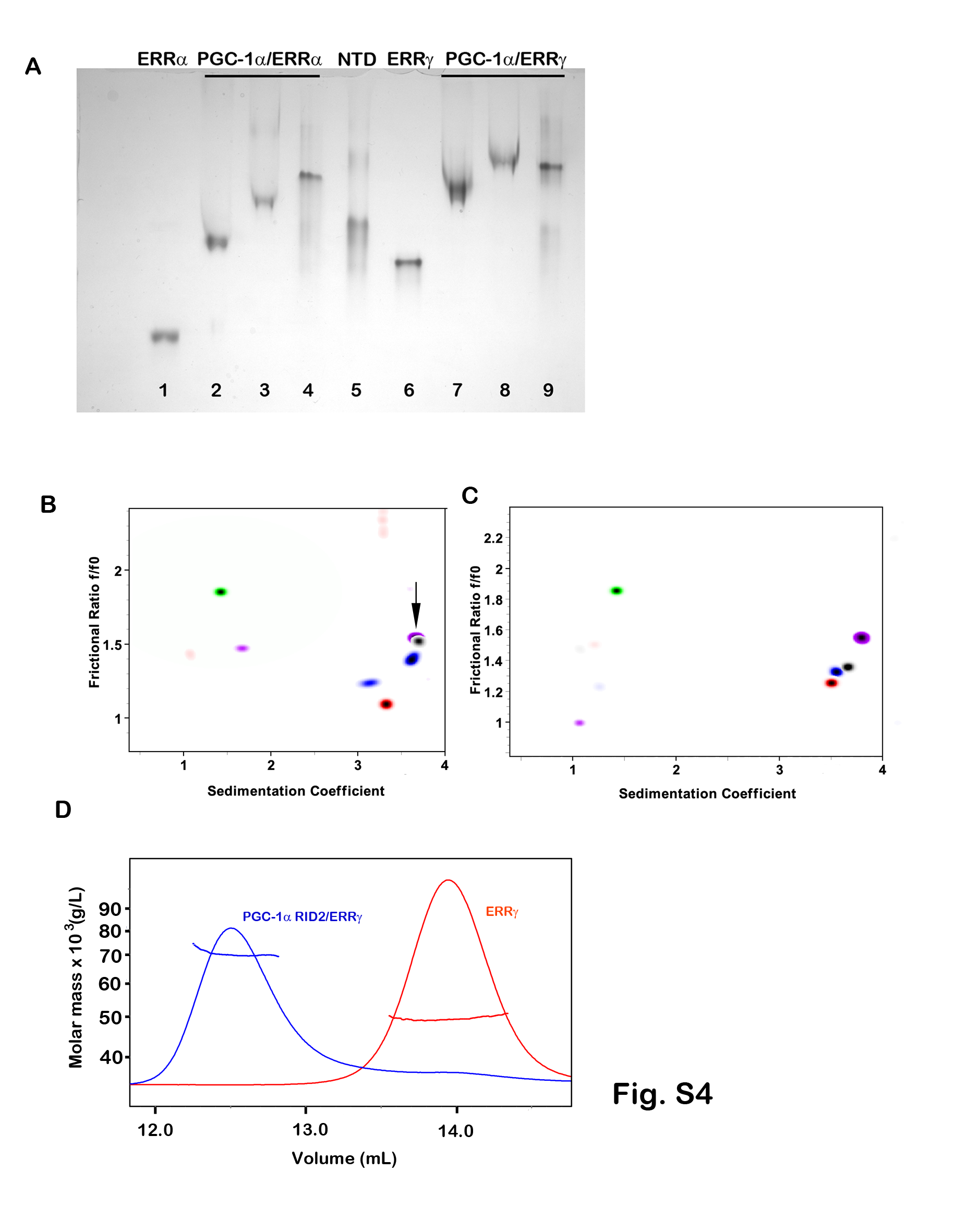

Supplement: Figure S4 — (TIF) [file pone.0067810.s004.tif]

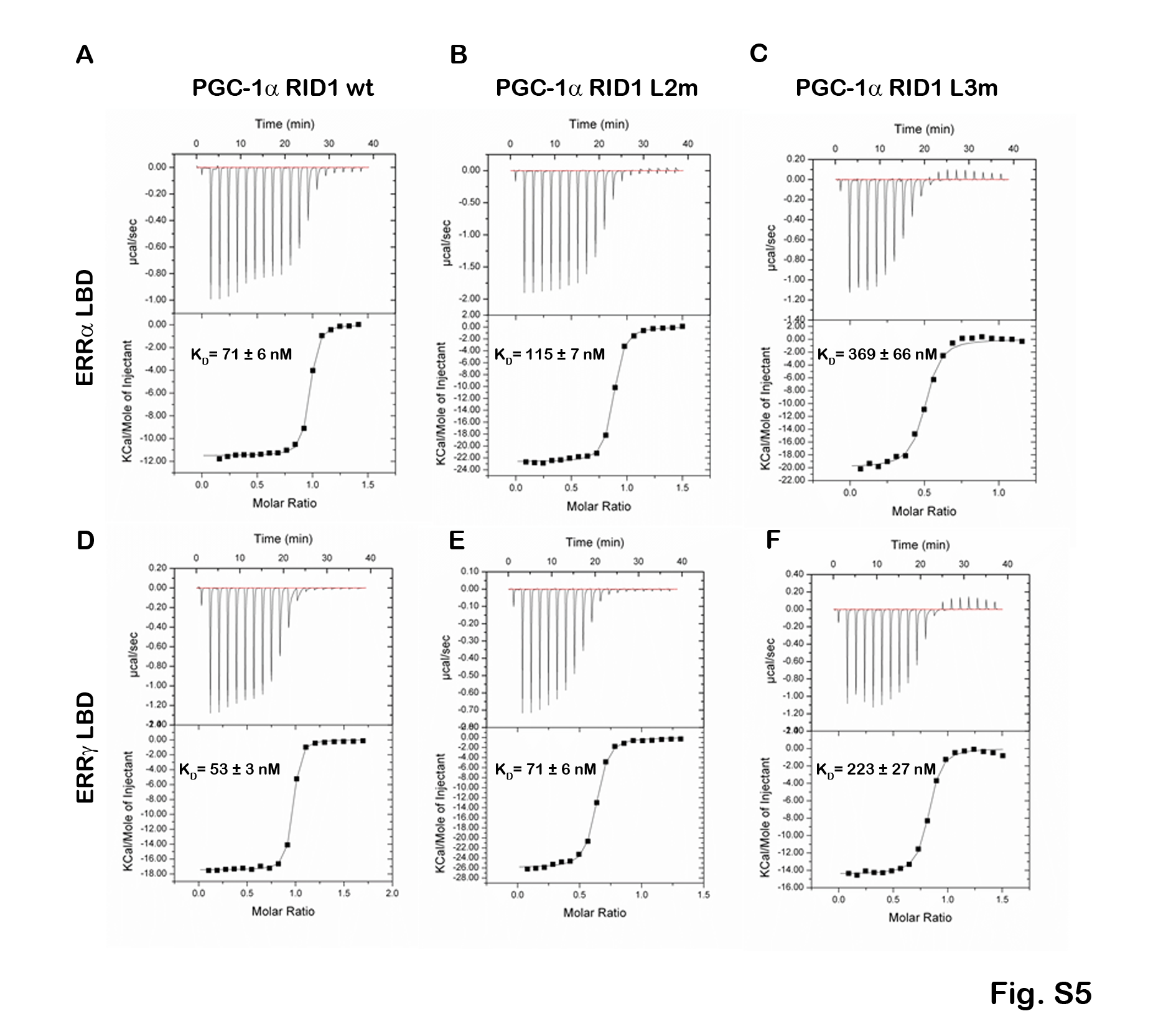

Supplement: Figure S5 — (TIF) [file pone.0067810.s005.tif]

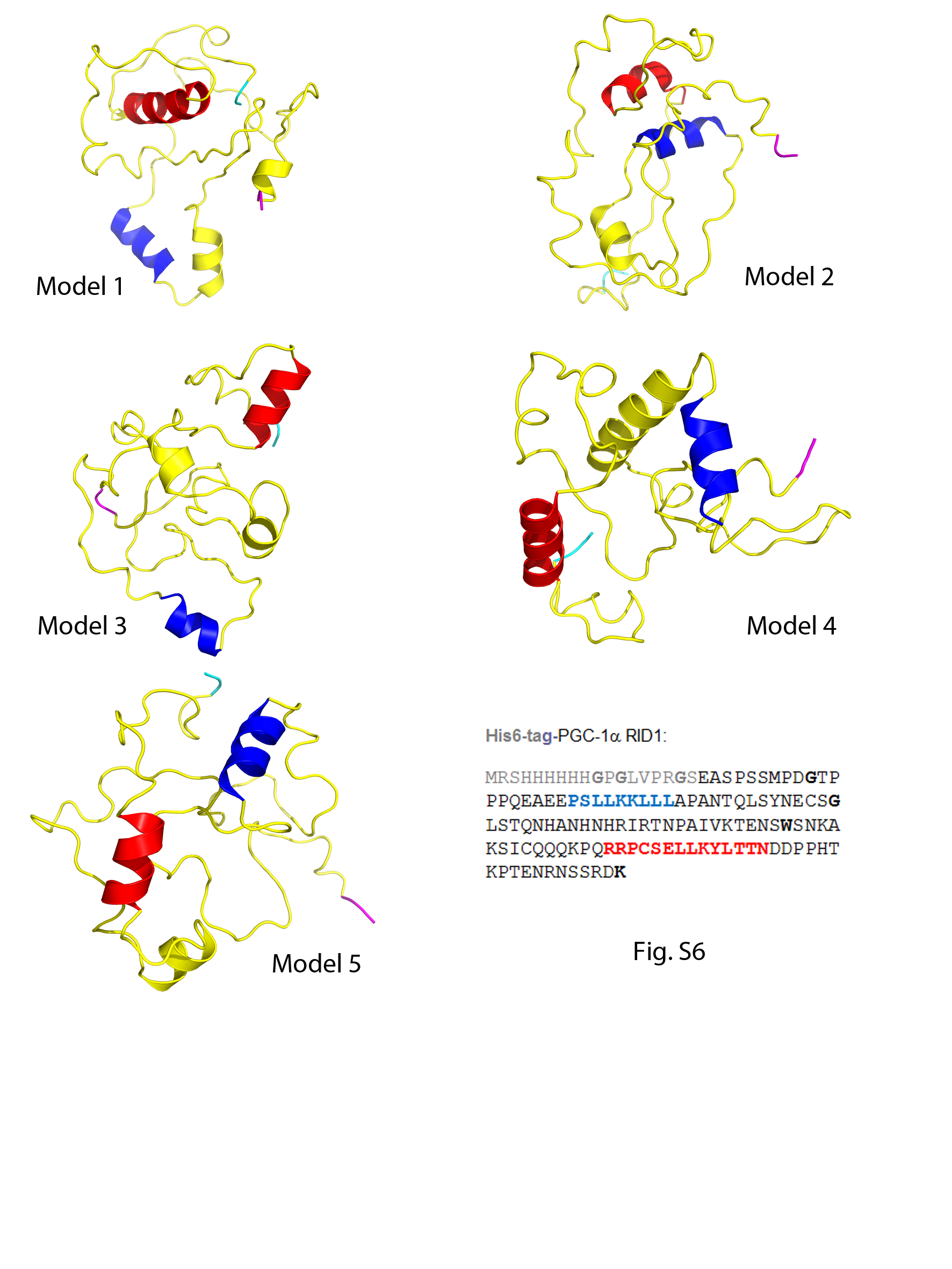

Supplement: Figure S6 — (TIF) [file pone.0067810.s006.tif]
