# Supplementary material for: The Asymmetric Binding of PGC-1α to the ERRα and ERRγ Nuclear Receptor Homodimers Involves a Similar Recognition Mechanism
Source: PLoS One. 2013 Jul 9;8(7):e67810. doi: 10.1371/journal.pone.0067810 (PMC3706463; doi:10.1371/journal.pone.0067810)
Supplement: Table S1 — (DOCX) [file pone.0067810.s007.docx]

**Table S1. Biophysical parameters for PGC-1α RID isolated and in complex with ERRα LBD and ERRγ LBD**

|  |  |  | **AUC** | |  | |
| --- | --- | --- | --- | --- | --- | --- |
| **Sample** | **MW (Da)** ^a^ | **Vbar(cm^3^/g)^a^** | | **S_20,w_**^b^ **(calc.**^c^**)** | | **f/fo**^b^ **(calc.**^c,d^**)** |
| **PGC-1α RID1** | 14773 | 0.7118 | | 1.23 | | 2.12 (1.9^d^) |
| **PGC-1α RID2** | 20263 | 0.7151 | | 1.44 | | 2.03 (1.97^d^) |
| **PGC-1α NTD** | 33603 | 0.7143 | | 1.97 | | 2.08 (2.03^d^) |
| **ERRα LBD** | 51360 | 0.7495 | | 3.25 (3.73) | | 1.3 (1.2^c^) |
| **ERRγ LBD** | 52946 | 0.7493 | | 3.56 (3.79) | | 1.2 (1.19^c^) |
| **PGC-1α RID2/ERRα LBD** | 71623 | 0.7398 | | 3.69 | | 1.52 (1.61^d^) |
| **PGC-1α RID2/ERRγ LBD** | 73209 | 0.7399 | | 3.79 | | 1.55 (1.59^d^) |

^a^ Molecular weights (MW) and partial specific volume (Vbar) at 20°Ccomputed using Ultrascan II; the molecular weights of ERRα and ERRγ are given for the dimer

^b^ experimental value

^c^ computed using SOMO hydrodynamics calculations [42-44] using the crystal structures of ERRα and ERRγ (pdb entries 3D24 and 1KV6, respectively), where the peptide coordinates were removed from the structures

^d^ computed using the calculated molecular weight, the calculated partial specific volume and the measured sedimentation coefficient S_20,w_
