# Supplementary material for: The Asymmetric Binding of PGC-1α to the ERRα and ERRγ Nuclear Receptor Homodimers Involves a Similar Recognition Mechanism
Source: PLoS One. 2013 Jul 9;8(7):e67810. doi: 10.1371/journal.pone.0067810 (PMC3706463; doi:10.1371/journal.pone.0067810)
Supplement: Table S2 — (DOCX) [file pone.0067810.s008.docx]

**Table S2. Thermodynamic parameters from isothermal titration calorimetry (ITC) of the interaction between ERRα or ERRγ and PGC-1α RID1 wt and LxxLL mutants**

| **ERR LBD** | **PGC-1α RID1** | **K_D_**  **(nM)** | **ΔH (kcal/mol)** | **ΔS**  **(cal/mol/°)** | **N** |
| --- | --- | --- | --- | --- | --- |
| **ERRα** | **Wild type** | 71 ± 6 | -11.5 ± 0.04 | - 6.52 | 0.940 ± 0.001 |
|  | **L2m** | 115 ± 7 | -22.65 ± 0.07 | - 45.5 | 0.846 ± 0.001 |
|  | **L3m** | 369 ± 66 | -19.92 ± 0.29 | - 38.5 | 0.483 ± 0.004 |
|  | **L2mL3m** | No binding | NA | NA | NA |
| **ERRγ** | **Wild type** | 53 ± 3 | - 17.44 ± 0.05 | -26.2 | 0.932 ± 0.001 |
|  | **L2m** | 71 ± 6 | - 25.93 ± 0.13 | -55.8 | 0.605 ± 0.001 |
|  | **L3m** | 223 ± 27 | - 14.43 ± 0.11 | - 18.8 | 0.793 ± 0.003 |
|  | **L2mL3m** | No binding | NA | NA | NA |

Data determined at 20°C and at pH=7.5 as described in *SI*.

NA: not applicable
