# Supplementary material for: The Asymmetric Binding of PGC-1α to the ERRα and ERRγ Nuclear Receptor Homodimers Involves a Similar Recognition Mechanism
Source: PLoS One. 2013 Jul 9;8(7):e67810. doi: 10.1371/journal.pone.0067810 (PMC3706463; doi:10.1371/journal.pone.0067810)
Supplement: Table S3 — (DOCX) [file pone.0067810.s009.docx]

**Table S3. Structural parameters of isolated PGC-1α RIDs from SAXS analysis**

| **Sample** | **R_g_(Å)**# | **D_max_(Å)**# | **Calc. Rg (Å) ^$^** | **Calc.Rg IDPs (Å)^&^** | **Lmax(Å)^%^** | **L(Å) ^%^** | **b(Å) ^%^** | **R_c_(Å) ^%^** |
| --- | --- | --- | --- | --- | --- | --- | --- | --- |
| **PGC-1α RID1 (132 aa)** | 36.2 | 132.5 | 15.3 | 32.5 | 474 | 406.1 | 22.4 | 3.6 |
| **PGC-1α RID2 (182 aa)** | 44.7 | 180 | 16.9 | 38.4 | 653 | 516.2 | 24.9 | 3.7 |
| **PGC-1α NTD (303 aa)** | 55.9 | 220 | 18.1 | 50.1 | n.d. | n.d. | n.d. | n.d. |

# from GNOM analysis

$ for globular proteins

& Rg ≈ 2.54(n)^0.522^

^%^Lmax= maximum physically extension of the protein; L=contour length of the chain; b=length of statistical element; Rc=radius of gyration of the cross section (see SI)
