# Supplementary material for: The Asymmetric Binding of PGC-1α to the ERRα and ERRγ Nuclear Receptor Homodimers Involves a Similar Recognition Mechanism
Source: PLoS One. 2013 Jul 9;8(7):e67810. doi: 10.1371/journal.pone.0067810 (PMC3706463; doi:10.1371/journal.pone.0067810)
Supplement: Table S4 — (DOCX) [file pone.0067810.s010.docx]

**Table S4. Parameters of the CRYSOL fits for ERR and PGC-1α RID/ERR complexes**

| **Data** | **Chi** | **Dro** | **Ra** | **RgE** | **RgT** |
| --- | --- | --- | --- | --- | --- |
| **ERRγ LBD** | 0.7 | 0.030 | 1.44 | 24.6 | 24.1 |
| PGC1αRID1/ERRαLBD | 1.3 | 0.063 | 1.40 | 30.1 | 30.3 |
| PGC-1αRID1/ERRγLBD | 0.9 | 0.030 | 1.40 | 29.5 | 29.5 |
| PGC-1αRID2/ERRγ LBD | 1.0* |  |  |  |  |
| PGC-1αNTD/ERRγ LBD | 1.1* |  |  |  |  |

* No Crysol fit. Chi for *ab initio* model shown.
