# Supplementary material for: The Asymmetric Binding of PGC-1α to the ERRα and ERRγ Nuclear Receptor Homodimers Involves a Similar Recognition Mechanism
Source: PLoS One. 2013 Jul 9;8(7):e67810. doi: 10.1371/journal.pone.0067810 (PMC3706463; doi:10.1371/journal.pone.0067810)
Supplement: File S1 — (DOCX) [file pone.0067810.s011.docx]

**Supplementary Information to the manuscript by Takacs et al**

**i/ Analysis of the SAXS data**

Small-angle X-ray scattering (SAXS) was used to gain insight into structural parameters of the PGC-1α RID in solution, either isolated or in complex with ERRα LBD and ERRγ LBD. From scattering data, a Guinier analysis can be performed at very low scattering angles, where a linear relationship is predicted between ln(I) and q^2^ for an ideal solution (where the scattering vector *q = 4π sin(θ)/λ*, *2θ* the scattering angle, *λ* the X-ray wavelength and I(q) the scattering intensity), thus allowing the corresponding radius of gyration, R_g_, to be derived.

**ii/ Analysis of the conformations of PGC-1α RID1 and RID2 by SAXS**

*Gyration radius calculation*

For the analysis of extended proteins, the gyration radius R_g_ should be calculated from the distance pair-distribution function P(r) which is more precise approach than the Guinier approximation [1].

$R_{g}^{2}=\frac{\int_{0}^{D} r^{2}P(r)dr}{2\int_{0}^{D} P\left( r \right)dr}$(1)

For globular proteins, an approximate value of the radius of gyration is given by the relationship *R_g_* ≈ 3(n)^1/3^ with n is the number of residues in the polypeptide chain [2]. On the other hand, for intrinsically disordered proteins, the radius of gyration follows the relationship Rg ≈ 2.54 n^0.522^ [3].

*Chain models*

To determine chain conformation and to find a good model that fit the data, the experimental curves are examined using the Kratky-Porod representation, which enhances the high q part of the experimental spectra. The Debye equation, used as reference, corresponds to the analytical expression for a freely jointed chain and giving a plateau at high q values

${I(q)}/{I\left( 0 \right)=2(\exp\left( -x \right)+x-1)/x^{2}}$ with x = q²R_g_² (2)

However, for a correct analysis of the data of isolated PGC-1α RIDs, the rigidity of the peptide bond must be taken into account, by using the model of Sharp and Bloomfield [4]. This model introduces a persistence length which accounts for local rigidity and allows characterizing the conformation by introducing two fitting parameters, b, the length of the statistical element, and L, the contour length of the chain, which are related by L=Nb, where N is the number of statistical elements:

${{I(q)}_{SB}}/{I(0)}=g_{D}\left( x \right)+ \frac{L}{b}\left[ \frac{4}{15}+ \frac{7}{15x}-\left( \frac{11}{15}+\frac{7}{15x} \right)exp(-x) \right]$ (3)

where, x equal to q²Lb/6, and g_D_(x) is the Debye function given in [1].

Furthermore, the presence of lateral chains will affect the thickness of the polypeptide chain and this will translate into a finite R_c_, the radius of gyration of the cross section. The form factor is then expressed as [5] :

${I\left( q \right)}/{I\left( 0 \right)=I_{SB}}\left( q \right)exp\left( -\frac{q^{2}R_{c}^{2}}{2} \right)$ (4)

**iii/ Analysis of the NMR data**

To deduce the portions of the PGC-1α RID1 and PGC-1α RID2 sequence that directly interact with ERRα LBD or ERRγ LBD, we examined the cross-peaks of glycine, tryptophan and threonine residues that are easily identified in the ^1^H-^15^N HSQC spectra.

1. The cross-peaks of glycine residues are typically found at low ^15^N chemical shift values (i.e. near the top of the ^1^H-^15^N HSQC spectrum). Three (out of five) and four (out of six) glycine cross-peaks remain undiminished and unshifted in the spectra of their PGC-1α RID/ERR complexes. Three glycine residues are located in the N-terminal GPGLVPRG sequence of the His_6_-tag that precedes the PGC-1α RID sequences (Fig. 1), while the additional 50 amino acids in PGC-1α RID2 contain just one glycine residue. It is likely that three of the remaining glycine cross-peaks belong to the N-terminal tag sequence and that, in the spectrum of PGC-1α RID2, the additional cross-peak belongs to the residue in the PKGSP sequence near the C-terminal end (Fig. 1). It is reasonable to assign the two cross-peaks that are affected upon complex formation to Gly131 and Gly163.
2. The side-chain amines of tryptophan residues give cross-peaks at high ^1^H and ^15^N chemical shifts. A cross-peak at 130.3 ppm (^15^N chemical shift) and 10.07 ppm (^1^H chemical shift) can therefore be assigned to the side-chain amine group of the sole residue of this type in the sequence, W189. This residue is broadened upon complex formation. C-terminal residues typically have narrower line-widths and have lower ^1^H chemical shifts and higher ^15^N chemical shifts. For both PGC-1α RIDs, the cross-peak most probably arising from the C-terminal residue (K235 for PGC-1α RID1 and S285 for PGC-1α RID2) remains, suggesting that neither C-terminus is affected by the binding to ERR LBD and, in particular, that K235 lies outside the binding region. The data thus indicate that binding involves the polypeptide chain encompassing G131, G163 and W189 and does not involve the N-terminal tag or the C-terminal residue.
3. Additional information can be obtained from the analysis of the region in which Thr/Ser cross-peaks are expected to lie, where 24 cross-peaks are expected and 24 are observed for PGC-1α RID1. On interaction with either ERR LBD 15 of these cross-peaks disappear and/or are shifted. Ten Thr/Ser residues are contained in the region G131-W189 (Fig. 1). To obtain five more, the interaction zone can be extended either towards the N-terminus, taking the SEASPSS motif (four Ser) adjacent to the N-terminal tag sequence or towards the C-terminus, thus encompassing the LLxYL motif L3 which is specific for interaction with ERRs [6-7]. This strongly suggests that the 5 additional cross-peaks originate from the C-terminal Thr/Ser residues and that the interaction domain extends as far as T216. A small number of PGC-1α RID1 cross-peaks are attenuated only on addition of ERRα LBD to PGC-1α RID2 (e.g. the serine/threonine cross-peak at 115.6/8.22). Perhaps binding of the C-terminal portion of PGC-1α RID1 is enhanced in some manner by the presence of the additional amino acids of PGC-1α RID2.

**Supplementary Methods**

**Cloning, protein expression and purification**

ERRα-189-423, PGC1α RID1 (122-235), RID2 (122-285) and NTD (1-285), as well as the LxxLL RID1 mutants L2m (LKKLL to LKKAA), L3m (LLKYL to AAKYL), and L2mL3m were cloned in pET24b and ERRγ 229-458 was cloned in pET15b. Proteins were expressed in *E. coli* BL21 (DE3) strain using Luria Bertani (ERRγ) and terrific broth (others) media. For labelling of PGC1α RID1 and RID2, expression was performed with *E. coli* BL21 (DE3) in M9 minimal medium containing ^15^NH_4_Cl. For purification, harvested cells were re-suspended in 20 mM Tris-HCl pH 8, 400 mM NaCl, 10% glycerol, 2 mM CHAPS, 5 mM imidazole and cOmplete, EDTA-free protease inhibitor cocktail tablet (Roche Applied Science), sonicated and centrifuged. The supernatant was loaded on 5 ml HisTrap FF crude column (GE Healthcare). The proteins were eluted at 250 mM imidazole and further purified by SEC on Superdex S75 (16/60 and 10/300, GE Healthcare) and Superdex S200 (16/60 and 10/300, GE Healthcare) columns using 20 mM Na-phosphate pH 6.5, 200 mM NaCl, 1% glycerol, 2 mM CHAPS buffer for NMR measurements and 20 mM Tris-HCl pH 8.0, 200 mM NaCl, 1% glycerol, 2 mM CHAPS buffer for other measurements. Protein samples were concentrated using Amicon-Ultra centrifugal filter units (Millipore). For SAXS, the His_6_-tag of ERR was cleaved using thrombin followed by a further SEC step. Purity and homogeneity of the protein were assessed by SDS-PAGE and complex formation was monitored by native PAGE.

**Tris/CAPS native PAGE**

The proteins were run on a polyacrylamide gel with a 8% separating and a 4% stacking part at 2 W constant power after 40 min pre-run of the unloaded gel. The Tris/CAPS buffer system contained 60 mM Tris base and 40 mM CAPS (3-cyclohexil-amino-1-propane-sulfonic acid), pH 9.4. About 5 µg protein was loaded per lane.

**Microscale Thermophoresis**

The microscale thermophoresis (MST) method is described in [8]. The apparent *K*_D,1_ and *K*_D,2_ values for the binding of one, respectively two molecules of PGC-1α RID1 to the ERR homodimer were measured using the Monolith NT 115 from NanoTemper Technologies GmbH. One of the binding partner (either the wild-type and mutant PGC-1α RID1 or the ERR LBD) were fluorescently labeled with the fluorescent dye NT-647 (NanoTemper Technologies) using the Monolith NT^TM^ Protein Labeling kit (amine reactive). The labeling procedure and the subsequent removal of free dye were performed within 1 hour. The solution of unlabeled PGC-1α RID1 (wild-type or LxxLL L2m and L3m mutants) was serially diluted from a concentration of 100-400 µM down to 1-10 nM in the presence of 20-50 nM labeled receptor. Alternatively, a solution of unlabeled ERR LBD was serially diluted from a concentration of about 35 µM down to 1 nM in the presence of 25 nM labeled PGC-1α RID1. Both experimental setups are equivalent for measuring the binding of one PGC-1α RID1 molecule to the ERR LBD (with binding affinity constant K_D,1_). The samples were loaded into hydrophilic capillaries (NanoTemper Technologies ref:K004). Measurements were performed at 20°C in 20 mM Hepes buffer, pH=7.4 with 100 mM NaCl, 1% glycerol, 1 mM CHAPS, 10 mM TCEP and 1 % BSA, by using 50% LED power and 80% IR-laser power. Data were analyzed using NanoTemper Analysis software, v.1.4.23.

**Isothermal titration calorimetry (ITC)**

Isothermal titration calorimetry experiments were performed using a MicroCal iTC200 (GE Healthcare) microcalorimeter thermostated at 20°C. The proteins were purified as described and dialyzed against 20mM Hepes pH 7.5, 120 mM NaCl, 0.2 % glycerol buffer. The PGC-1a RID wild-type and LxxLL mutants (340-580 µM) were injected into a 40-70 μM ERR solution (200-μl sample cell). Each titration series consisted of an initial injection of 0.4 µl and was followed by 19 injections of 2 µl. Data was analyzed with the software Origin 7.0 (OriginLab) using the one set of sites model. Standard free energies of binding and entropic contributions were obtained, respectively, as ΔG = −RT ln(K_a_) and TΔS = ΔH − ΔG from the K_a_ and ΔH values derived from ITC curve fitting.

**Small Angle X-ray Scattering**

Synchrotron X-ray solution scattering data were collected at the X33 beamline (DESY, Hamburg) [9] using PILATUS detector at a sample-detector distance of 2.7 m, covering the range of momentum transfer 0.01 < *q* < 0.6 Å^-1^ (*q = 4π sin(θ)/λ* where *2θ* is the scattering angle and *λ* = 0.15 nm is the X-ray wavelength) in eight frames (15 seconds each) to check for possible radiation damage. All scattering measurements were carried out at 10°C using automated filling [10] and samples were measured at several solute concentrations.

SAXS data were also collected at the SWING beamline at SOLEIL Synchrotron (Gif-sur-Yvette, France), using a 17 x 17 cm2 low-noise Aviex CCD detector positioned at a distance of 2.107 m from the sample. Sample solutions were circulated in a thermostated Quartz capillary with a diameter of 1.5 mm and 10µm wall thickness, positioned within a vacuum chamber. About 20-40 µL protein were injected at relatively high concentrations (4 to 10 mg/ml) into a SEC Column (SHODEX KW402.5), using an Agilent © HPLC system and eluted directly into the SAXS flow-through capillary cell at a flow rate of 150 µl/min [11]. SAXS data were collected online throughout the whole elution time, with 2 s frame duration and 1 s dead time between frames. In total 250 frames were collected, normalized to transmitted intensity. The frames corresponding to the elution peak of the complex were averaged using “Foxtrot”, a dedicated home-made application.

The data were processed using standard procedures in the program package PRIMUS [12]. The forward scattering *I(0)* and the radii of gyration *R_g_* were evaluated using the Guinier approximation, assuming that at very small angles (*q < 1.3/R_g_*), the intensity is represented as *I(q) = I(0) exp(-(qR_g_)^2^/3)*. The maximum dimensions *D_max_* were computed using the indirect transform package GNOM [13] which also provides the distance distribution functions *p(r)*. Low resolution shape analysis of the solutes was performed using the ab initio program DAMMIF [14] which represents the macromolecule by an assembly of densely packed beads and employs simulated annealing (SA) to build a compact interconnected configuration of beads that fits the experimental data *I_exp_(q)*. The scattering from the atomic models was calculated using the program CRYSOL [15] which either predicts theoretical scattering patterns or fits the experimental data by adjusting the excluded volume and the contrast of the hydration layer. The program SASREF [16] was employed for molecular rigid body modeling of the PGC-1α RID1/ERR complex, based on the crystal structure of ERRγ LBD [17] and an atomic model of PGC-1α RID1 *de novo* predicted by Robetta server [18]. SASREF uses SA to position the domains with respect to each other, forming an interconnected assembly without steric clashes, while minimizing the discrepancy between the experimental data and the computed scattering profile. Distance restraints were applied to ensure the contact between motif L3 and the coactivator groove of the LBD. For both ab initio and rigid-body analysis, multiple runs were performed to verify the stability of the solution, and the most typical reconstructions were selected using the programs DAMAVER [19] and SUPCOMB [20]. The latter program aligns two arbitrary low or high resolution models represented by ensembles of points by minimizing a dissimilarity measure called normalized spatial discrepancy.

**Analytical Ultracentrifigation**

Experiments were performed using a Beckman Proteomelab XL-I ultracentrifuge (Beckman Instruments, CA, USA) with an An50Ti 8-hole rotor fitted with epon-charcoal-filled 1.2 cm path-length two-sector centerpieces for sedimentation velocity with sapphire windows. Velocity sedimentation was monitored at 4ºC in either absorbance or intensity mode. The samples were spun between 38 and 50 krpm until the sample had fully sedimented. The absorbance of the samples was set to 0.35-0.45 at 280 nm. The data were analyzed using the UltraScan software version 9.9 [21,22] All datasets were initially fitted with the 2-dimensional spectrum analysis with simultaneous time- and radially invariant noise removal and meniscus fitting [23-24]. The resulting data were refined by parsimonious regularization [25] with the genetic algorithm analysis [26]. Statistics were determined by Monte Carlo analysis [27] in combination with the genetic algorithm analysis. Partial specific volumes were calculated from protein sequence as implemented in UltraScan.

**Size-Exclusion Chromatography coupled to Multi-Angle and Quasi-Elastic Light Scattering**

SEC-MALS/QELS experiments were performed on a multi-angle light scattering detector (miniDAWN TREOS, Wyatt Technologies) coupled in-line with SEC and an interferometric refractometer (Optilab T-rEX, Wyatt Technologies). A Superdex S200 10/300 GL column (total volume 24 mL, GE Healthcare) or alternatively an Agilent Bio SEC-3 column (4.6 mL total volume, Agilent Technologies) with a flow rate of 0.5 and 0.3 mL/min, respectively, were used to separate the sample before performing the MALS/QELS measurement. Experiments were done with 50-90 µL protein sample at concentrations 1-3 mg/mL in 25 mM Hepes pH 7.4, 100 mM NaCl. For the determination of R_h_, measurements were first performed on standard globular proteins (BioRad), RNAse (13.7 kDa), carbonic anhydrase (29 kDa), ovalbumin (42.8 kDa), bovin serum albumin (66.4 kDa) and conalbumin (75 kDa) using a Wyatt QELS Detector embedded in the miniDAWN TREOS instrument that measures the dynamic light scattering signal and calculates the autocorrelation function and the corresponding R_h_. For the MALS experiments, the molar mass was determined by construction of Debye plot using Zimm formalism (plot of K*c/R(θ) as a function of sin^2^(θ/2)) at 0.5 and 1 sec data interval, for Agilent Bio-SEC-3 and Superdex S200 column, respectively. The analysis of the data was performed using the ASTRA 5.3.4 software (Wyatt Technologies).

**Electrospray Ionization Mass Spectrometry**

Prior to ESI-MS analysis, samples were desalted on NAP-5 desalting column (GE Healthcare) in 200 mM ammonium acetate (pH 7.4). ESI-MS measurements were performed on an ESI-TOF mass spectrometer (MicrOTOF, Bruker Daltonic, Germany). Purity and homogeneity of ERR were verified by MS analysis in denaturing conditions. The non-denaturing mass measurements of the non-covalent complexes were performed in ammonium acetate (200 mM; pH 7.4). Samples were diluted to 6 pmol/μl in ammonium acetate buffer and continuously infused into the ESI ion source at a flow rate of 3 μl/min through a Harvard syringe pump (Harvard Apparatus model 11). Interface conditions were optimized to obtain optimum sensitivity and spectrum quality, while preventing dissociation of the complex. In particular, the capillary exit (CE) controlling the kinetic energy of the ions and the transfer-/storage-time related to the hexapole storage was set to 150V.

**Molecular dynamics simulation**

Prior to MD simulations, H atoms were added to the model of PGC1-α RID1/ERRγ dimer, using the HBUILD facility [28] in the CHARMM program [29]. Implicit solvent MD were performed on the complex using the generalized Born implicit solvent model FACTS [30] in CHARMM, with a solute dielectric constant of 1.0 and an integration time step of 2 fs. The system was energy minimized using 300 steps of Steepest Descent, followed by 200 steps of Conjugate Gradient. Two series of MD simulations were performed at 350 and 500 K, consisting of heating over 20 ps, equilibration for 20 ps followed by short production runs of 100 ps. The LBD and motif L3 were constrained to their positions as found in the crystal structure 1KV6, and motif L2 was maintained in α-helical conformation. Harmonic constraints were used with a force constant of 10 kcal/mol. In order to obtain two conformation ensembles with either small or larger conformational changes with respect to the initial structure, a first group of structures were extracted from the equilibration step at 350 K and a second one from the production step at 500 K.

**Figure preparation**

The figures of the envelopes and the solution structures were made using Pymol 1.4.1, Chimera 1.5.3 and the plots were drawn by using Sigmaplot 11.

**References**

1. Perez J, Vachette P, Russo D, Desmadril M, Durand D (2001) Heat-induced unfolding of neocarzinostatin, a small all-protein investigated by small-angle X-ray scattering. J Mol Biol 308:721-743.

2. Narang P, Bhushan K, Bose S, Jayaram B (2005) A computational pathway for bracketing native-like structures fo small alpha helical globular proteins. Phys Chem Chem Phys 7:2364-2375.

3. Bernado P, Blackledge M (2009) A Self-Consistent Description of the Conformational Behavior of Chemically Denatured Proteins from NMR and Small Angle Scattering. Biophys J 97:2839:2845.

4. Sharp P, Bloomfield V (1968) Light scattering from wormlike chains with excluded volume effects. Biopolymers 6:1201-1211.

5. Rawiso M, Duplessix R, Picot C (1987) Scattering function of polystyrene. Macromolecules 20:630-648.

6. Huss JM, Kopp RP, Kelly DP (2002) Peroxisome Proliferator-activated Receptor Coactivator-1alpha (PGC-1alpha ) Coactivates the Cardiac-enriched Nuclear Receptors Estrogen-related Receptor-alpha and -gamma . IDENTIFICATION OF NOVEL LEUCINE-RICH INTERACTION MOTIF WITHIN PGC-1alpha. J Biol Chem 277:40265-40274.

7. Gaillard S, Dwyer MA, McDonnell DP (2007) Definition of the molecular basis for estrogen receptor-related receptor-alpha-cofactor interactions. Mol Endocrinol 21:62-76.

8. Jerabek-Willemsen M, Wienken CJ, Braun D, Baaske P, Duhr S (2011) Molecular interaction studies using microscale thermophoresis. Assay Drug Dev Technol 9:342-353.

9. Roessle M, Klaering R, Ristau U, Robrahn B, Jahn D, et al (2007) Upgrade of the small-angle X-ray scattering beamline X33 at the European Molecular Biology Laboratory, Hamburg. J Appl Crystallogr 40:s190-s194.

10. Round A, Franke D, Moritz S, Huchler R, Fritsche M, et al (2008) Automated sample-changing robot for solution scattering experiments at the EMBL Hamburg SAXS station X33. J Appl Crystallogr 41:913-917.

11. David G, Perez J (2009) Combined sampler robot and high-performance liquid chromatography: a fully automated system for biological small-angle X-ray scattering experiments at the Synchrotron SOLEIL SWING beamline. J Appl Crystallogr 42:892-900.

12. Konarev P, Ward B, Sokolova A, Koch MH, Svergun DI (2003) PRIMUS -a Windows-PC based system for small-angle scattering data analysis. J Appl Crystallogr 36:1277-1282.

13. Svergun DI (1992) Determination of the regularization parameter in indirect-transform methods using perceptual criteria. J Appl Crystallogr 25:495-503.

14. Franke D, Svergun D (2009) DAMMIF, a program for rapid ab-initio shape determination in small-angle scattering. J Appl Crystallogr 42:342-346.

15. Svergun DI, Barberato C, Koch MH (1995) CRYSOL - a program to evaluate X-ray solution scattering of biological macromolecules from atomic coordinates. J Appl Crystallogr 28:768-773.

16. Petoukhov MV, Svergun DI (2005) Global rigid body modelling of macromolecular complexes against small-angle scattering data. Biophys J 89:1237-1250.

17. Greschik H, Wurtz J-M, Sanglier S, Bourguet W, van Dorsselaer A, et al (2001) Structural and functional evidence for ligand-independent transcriptional activation by the estrogen-related receptor 3. Mol Cell 9:303-313.

18. Kim DE, Chivian D, Baker D (2004) Protein structure prediction and analysis using the Robetta server. Nucleic Acids Res 32:W526-W531.

19. Volkov V, Svergun DI (2003) Uniqueness of ab initio shape determination in small angle scattering. J Appl Crystallogr 36:860-864.

20. Kozin M, Svergun DI (2001) Automated matching of high- and low-resolution structural models. J Appl Crystallogr 34:33-41.

21. Demeler B, Brookes E, Cao W, Dubbs B, Zollars D, et al (2010) UltraScan-II version 9.9.

22. Demeler B (2005) UltraScan - A Comprehensive Data Analysis Software Package for Analytical Ultracentrifugation Experiments, in Modern Analytical Ultracentrifugation: Techniques and Methods, eds Scott D, Harding S, Rowe A (Royal Society of Chemistry, Cambridge), pps. 210-229.

23. Brookes E, Boppana R, Demeler B (2006) Computing Large Sparse Multivariate Optimization Problems with an Application in Biophysics. Supercomputing '06 ACM0-7695-2700-0/06.

24. Brookes E, Cao W, Demeler B (2010) A two-dimensional spectrum analysis for sedimentation velocity experiments of mixtures with heterogeneity in molecular weight and shape. Eur Biophys J 39:405-414.

25. Brookes E, Demeler B (2007) Parsimonious Regularization using Genetic Algorithms Applied to the Analysis of Analytical Ultracentrifugation Experiments. GECCO Proceedings ACM 1978-1-59593-697-4/07/0007.

26. Brookes E, Demeler B (2006) Genetic Algorithm Optimization for obtaining accurate Molecular Weight Distributions from Sedimentation Velocity Experiments in Analytical Ultracentrifugation VIII, Progress in Colloid Polym. Sci, eds Wandrey C, Colfen H (Springer, New York) pps. 78-82.

27. Demeler B, Brookes E (2008) Monte Carlo analysis of sedimentation experiments. Colloid Polym Science 286:129-137

28. Brunger AT, Karplus M (1988) Polar hydrogen positions in proteins: empirical energy placement and neutron diffraction comparison. Proteins 4:148-156.

29. Brooks BR, Bruccoleri RE, Olafson BD, States DJ, Swaminathan S, Karplus M (1983) CHARMM: A program for macromolecular energy, minimization and dynamics calculations. J Comp Chem 4:187-217.

30. Haberthur U, Caflisch A (2008) FACTS: Fast analytical continuum treatment of solvation. J Comput Chem 29:701-715.

31. Plewniak F, Bianchetti L, Brelivet Y, Carles A, Chalmel F, et al (2003) PipeAlign: a new toolkit for protein family analysis. Nucl Acids Res 31:3829-3832.

32. Waterhouse AM, Procter JB, Martin DMA, Clamp M, Barton GJ (2009) Jalview Version 2-a multiple sequence alignment editor and analysis workbench. Bioinformatics 25:1189-1191.

33. Vacic V, Uversky V, Dunker AK, Lonardi S (2007) Composition Profiler: a tool for discovery and visualization of amino acid composition differences. BMC Bioinformatics 8:211.

34. Lieutaud P, Canard B, Longhi S (2008) MeDor: a metaserver for predicting protein disorder. BMC Genomics 9 Suppl 2:S25.

35. Dosztanyi Z, Csizmok V, Tompa P, Simon I (2005) IUPred: web server for the prediction of intrinsically unstructured regions of proteins based on estimated energy content. Bioinformatics 21:3433-3434.

36. Linding R, Russell RB, Neduva V, Gibson TJ (2003) GlobPlot: exploring protein sequences for globularity and disorder. Nucl Acids Res 31:3701-3708.

37. Linding R, Jensen LJ, Diella F, Bork P, Gibson TJ, Russell RB 2003 Protein disorder prediction: implications for structural proteomics. Structure 11:1453-1459.

38. Prilusky J, Felder CE, Zeev-Ben-Mordehai T, Rydberg EH, Man O, et al (2005) FoldIndex: a simple tool to predict whether a given protein sequence is intrinsically unfolded. Bioinformatics 21:3435-3438.

39. Yang ZR, Thomson R, McNeil P, Esnouf RM (2005) RONN: the bio-basis function neural network technique applied to the detection of natively disordered regions in proteins. Bioinformatics 21:3369-3376.

40. Receveur-Brechot V, Bourhis JM, Uversky VN, Canard B, Longhi S (2006) Assessing protein disorder and induced folding. Proteins 62:24-45.

41. Boze H, Marlin T, Durand D, Perez J, Vernhet A, et al (2010) Proline-Rich Salivary Proteins Have Extended Conformations. Biophys J 99:656-665.

42. Brookes E, Demeler B, Rocco M (2010) Developments in the US-SOMO Bead Modeling Suite: New Features in the Direct Residue-to-Bead Method, Improved Grid Routines, and Influence of Accessible Surface Area Screening. Macromol Biosci 10:746-753.

43. Brookes E, Demeler B, Rosano C, Rocco M (2010) The implementation of SOMO (SOlution MOdeller) in the UltraScan analytical ultracentrifugation data analysis suite: enhanced capabilities allow the reliable hydrodynamic modeling of virtually any kind of biomacromolecule. Eur Biophys J 39:423-435.

44. Rai N, Nollmann M, Spotorno B, Tassara G, Byron O, Rocco M (2005) SOMO (SOlution MOdeler) differences between X-Ray- and NMR-derived bead models suggest a role for side chain flexibility in protein hydrodynamics. Structure 13:723-734.

**Supplementary Figure Legends**

**Figure Supp.1:** **Multiple sequence alignment of the NTD PGC-1α sequences found in recently diverged vertebrate species**. The position of the three NR boxes is indicated. The alignment was made by using the Pipealign suite [31] and represented using Jalview [32]. SwissProt accession numbers are: Q9UBK2 (human PGC-1α), O70343 (mouse PGC-1α), A9YWH5 (goat PGC-1α), Q865B6 (pig PGC-1α), D0VE08 (buffalo PGC-1α), F6YR50 (horse PGC-1α), E2QWA6 (dog PGC-1α), Q865B7 (bovin PGC-1α), Q60GU0 (chicken PGC-1α), **Q9QYK2 (rat** PGC-1α).

**Figure Supp.2:** **In silico analysis of the PGC-1α NTD sequence composition and disorder predictions**. A. and B. Amino acid composition analysis was performed using composition profiler [33], available at <http://www.cprofiler.org>. Composition Profiler detects enrichment or depletion patterns of individual amino acids classified by several physico-chemical and structural properties. The query PGC-1α NTD sequence was compared to a reference proteins database which provides the background amino acid distribution.

A. The comparison with the reference PDB S25 dataset which represents proteins which are easy to crystallize and possess an ordered structure shows that the sequence of PGC-1α NTD is depleted in some order-promoting residues (Y, F, V, M, I). Moreover, there is significant enrichment in negatively charged residues and a marked abundance of amino acids P, S and N, considered to be markers of loops. B. The comparison with the reference DisProt 3.4 dataset which represents typical IDPs reveals that the PGC-1α NTD sequence is enriched in a few order-promoting amino acids, in particular in C and to a lesser extent in W and L. Bars above zero indicate the abundance of a given residue and the bars below zero indicate a deficiency of a given residue, relative to the reference proteins database.

C. Unified view of the outputs of disorder predictors and results of Hydrophobic Cluster Analysis (HCA). The disorder prediction for the N-terminal region (1-285) of the human PGC-1α (Q9UBK2) was performed using the MeDor metaserver [34]. α-helices are drawn in red, β-strands are represented by blue arrows. The PGC-1α NTD is predicted to contain large unstructured segments by algorithms such as IUPred [35], Globplot [36], DisEMBL [37], FoldIndex [38] and RONN [39], while hydrophobic cluster analysis (HCA) indicates clusters of hydrophobic residues at the very N-terminus of PGC-1α NTD and in the regions spanning motifs L1, L2 and L3. On the HCA plot clusters of hydrophobic residues suggest potential secondary structure or potentiality to fold upon interaction with appropriate partner. Predicted disordered regions are represented by bidirectional arrows of different colours for different predictor algorithms, IUPred [35], Globplot [36], DisEMBL [37], FoldIndex [38] and RONN [39].

**Figure S3:** **Hydrodynamic properties of isolated PGC-1α and its biochemical and structural characterization.** **A**. The hydrodynamic radius (Rh) was measured by size-exclusion chromatography (SEC) coupled to a multi-angle and quasi-elastic light scattering (MALS/QELS) apparatus for PGC-1α RID1, RID2 and NTD constructs (red squares) and for well characterized globular proteins of different masses as a function of molecular weight (blue diamonds). The investigated proteins each elute as a single peak and their measured molecular weights are in agreement with the calculated masses of the monomeric species. While the hydrodynamic radii (Rh) of globular proteins follow a linear behaviour as a function of molecular weight, those of PGC-1α RID1, RID2 and NTD particles depart markedly from a straight line and are significantly larger than values expected for globular proteins. **B**. SDS-PAGE polyacrylamide gel for the purified PGC-1α RID1 (lane 2, 14.7 kDa), PGC-1α RID2 (lane 3, 20.2 kDa) and PGC-1α NTD (lane 4, 33.6 kDa) proteins, together with the molecular weight marker (lanes 1 and 5) showing the abnormal migration of the PGC-1α RID IDP. The apparent molecular weight are much larger than the computed values (Table S1). Atypical mobility in SDS-PAGE is a typical feature of IDPs [40]. **C-E.** The SAXS analysis of free PGC-1α RID constructs indicates the presence of partially structured regions (see Suppl. Info). **C**. Pair distance distribution functions P(r) for PGC-1α RID1 (red), PGC-1α RID2 (green) and PGC-1α NTD (blue). Also shown in the inset are the corresponding scattering spectra plotted using the same colors. For IDPs, the gyration radius R_g_ is calculated from the distance pair-distribution function P(r). **D**. Normalized Kratky-Porod representations for PGC-1α RID1 (red), PGC-1α RID2 (green) and PGC-1α NTD (blue). Kratky-Porod representations enhance the high q part of the experimental data [41]. For PGC-1α RID1 and RID2, the Kratky-Porod plots show similar behaviour as that expected for a freely jointed chain (discontinuous black line) with slight deviations from the plateau at the larger angles, suggesting the presence of short-range, sequence-local order. For PGC-1α NTD, the analysis suggests the presence of a partially structured part. Since PGC-1α NTD differs only by the N-terminus, it means that this part is partially structured or that it might affect the overall structure of the domain. **E**. Scattering from PGC-1α RID1 (red), PGC-1α RID2 (green) compared with the scattering curves calculated according to the Sharp-Bloomfield model with thickness model (black line) with the geometrical parameters shown in Table S3. The contour lengths of PGC-1α RID1 and RID2 L are shorter than L_max_, the maximal possible physical extension of the protein, suggesting the presence of short-range, sequence-local order. This result is further corroborated by the values obtained for b and R_c_, which are larger than the corresponding values of IDPs (b≈18-20 Å and R_c_ 2.4 Å) [41]. For the PGC-1α NTD, the Kratky-Porod plot decays faster than a freely jointed chain, suggesting the presence of a partially structured part and the analysis above cannot be applied. **F**. Kratky-Porod representation ERRγ LBD dimer (red) that is representative of a folded protein domain. (**G-I**) NMR of isolated PGC-1α RID1 and RID2. ^1^H-^15^N HSQC spectra of (F) PGC-1α RID1 and (G) PGC-1α RID2 showing little dispersion of resonances. Superposition of the two spectra (H) shows that the spectrum of RID2 (red) is essentially the same as that of RID1 (black) with additional peaks arising from the extra amino acids. Note that the folded cross-peak of a side-chain resonance at 7.12 ppm has different apparent ^15^N chemical shift values due solely to the use of different spectral widths in the two experiments.

**Figure S4: Biophysical characterization of the stoichiometry of the PGC-1α RID2/ERR complexes** **A**. Tris CAPS native polyacrylamide gel of the different proteins. Lane 1: ERRα and lanes 2-3-4: complexes between ERRα and PGC-1α RID1, RID2 and NTD, respectively. Lane 5: PGC-1α NTD. Lane 6: ERRγ and lanes 7-8-9: complexes between ERRγ and PGC-1α RID1, RID2 and NTD, respectively. **B** and **C**. Pseudo-3D plots for GA-MC analysis, showing the sedimentation coefficients and frictional ratios of isolated (**B**) ERRα and (**C**) ERRγ, PGC-1α RID2 and the respective titration series. The saturated complex is shown by an arrow. The color code is identical to that used for van Holde-Weischet plots (in Figs.3A-B). In the composite plots, the S_20,W_ and f/fo values of ERRα LBD and ERRγ LBD are in agreement with values calculated using US-SOMO, a bead modelling suite for macromolecule hydrodynamic calculations [42-44]. On interaction with PGC-1α RID2, both values increase when comparing the 1:0.5 to the 1:1.4 ratios and then saturate for molar ratios above 1:1.4. At the molar ratio 1:3, the excess of free PGC-1α RID2 appears in the spectrum at f/fo and S_20,W_ values similar to those of isolated PGC-1α. As expected, the frictional ratio f/fo of the PGC-1α RID2/ERR complex is larger than that of isolated ERR LBD, suggesting a more elongated entity than the ERR LBD. On the other hand, the frictional ratio f/fo of the PGC-1α RID2/ERR complex is smaller than the value of isolated PGC-1α RID2, suggesting a compaction of the molecule upon binding to ERR LBD. At a molar ratio of 1:0.5, the graph reveals two species with different S_20,W_ and f/fo values that are likely to be identified as free ERR LBD and PGC-1α RID2 bound ERR complex. **D.** SEC-MALS analysis of the isolated ERRγ LBD (red) and of the corresponding complex with PGC-1α RID2 (blue) on a Superdex S200 10/300 GF column at room temperature. The differential refractive index profiles are shown as a function of retention volume. The molar mass determined by MALS analysis is shown in the graph as a collection of colored circles superimposed on each SEC profile. The y axis denotes the molar mass in kDa.

**Figure Supp.5:** **Isothermal calorimetric analysis of the binding interaction between ERR LBD and PGC-1α RID1 wild-type or L2 and L3 mutants.** Isotherms are shown for the binding interaction between ERRα LBD (**A-C**) or ERRγ LBD (**D-F**) and PGC-1α RID1. Wild-type PGC-1α RID1 binds to ERRα LBD (**A**) and ERRγ LBD (**D**) with high affinity. Disrupting the LxxLL L2 motif by alanine mutations results in an decrease in the binding affinity to ERRα LBD (**B**) and ERRγ LBD (**E**). The alanine mutation of motif L3 results in an even larger decrease in the binding affinity of PGC-1α RID1 L3m for ERRα LBD (**C**) and ERRγ LBD (**F**). The thermodynamic parameters (dissociation constant (Kd), enthalpy (ΔH) and change in entropy (ΔS)) determined by ITC are presented in Table S2.

**Figure Supp.6:** **Secondary structure predictions for PGC-1α RID1**. Schematic cartoon representation of five different models of PGC-1α RID1 predicted by Robetta. The Cα-trace of the PGC-1α RID1 molecule is shown in yellow. The Robetta secondary structure analysis consistently predicts helices for the L2 and L3 RID motifs which are shown in blue and red, respectively. The N- and C-terminal parts of the PGC-1α RID1 construct are depicted in magenta and cyan, respectively. The amino acid sequence of His_6_-tagged PGC-1α RID1 is given and the residues belonging to the L2 and L3 helices are colored in the same colors as used for structure representation.
